# Alpha-Synuclein Aggregation Pathway in Parkinson’s Disease: Current Status and Novel Therapeutic Approaches

**DOI:** 10.3390/cells11111732

**Published:** 2022-05-24

**Authors:** Marija Vidović, Milena G. Rikalovic

**Affiliations:** 1 Laboratory for Plant Molecular Biology, Institute of Molecular Genetics and Genetic Engineering, University of Belgrade, Vojvode Stepe 444a, 11042 Belgrade, Serbia; 2 Environment and Sustainable Development, Singidunum Univeristy, Danijelova 32, 11010 Belgrade, Serbia; mrikalovic@singidunum.ac.rs

**Keywords:** α-synuclein oligomers and fibrils, disaggregators, high throughput anti-aggregation drug screening, intrinsically disordered proteins, protein misfolding, rationally designed peptidomimetics, structure/function relationship, synucleinopathies

## Abstract

Following Alzheimer’s, Parkinson’s disease (PD) is the second-most common neurodegenerative disorder, sharing an unclear pathophysiology, a multifactorial profile, and massive social costs worldwide. Despite this, no disease-modifying therapy is available. PD is tightly associated with α-synuclein (α-Syn) deposits, which become organised into insoluble, amyloid fibrils. As a typical intrinsically disordered protein, α-Syn adopts a monomeric, random coil conformation in an aqueous solution, while its interaction with lipid membranes drives the transition of the molecule part into an α-helical structure. The central unstructured region of α-Syn is involved in fibril formation by converting to well-defined, β-sheet rich secondary structures. Presently, most therapeutic strategies against PD are focused on designing small molecules, peptides, and peptidomimetics that can directly target α-Syn and its aggregation pathway. Other approaches include gene silencing, cell transplantation, stimulation of intracellular clearance with autophagy promoters, and degradation pathways based on immunotherapy of amyloid fibrils. In the present review, we sum marise the current advances related to α-Syn aggregation/neurotoxicity. These findings present a valuable arsenal for the further development of efficient, nontoxic, and non-invasive therapeutic protocols for disease-modifying therapy that tackles disease onset and progression in the future.

## 1. Introduction

Parkinson’s disease (PD) is progressive and the second-most prevalent neurodegenerative disorder after Alzheimer’s, sharing unclear pathophysiology and huge social costs. PD is tightly associated with the presence of α-synuclein (α-Syn) oligomers, organised into insoluble amyloid fibrils. Their neurotoxicity is related to membrane integrity loss and prion-like ‘neuron-to-neuron’ disease spreading. Despite a huge body of scientific data and investments over decades, the cure for this disease has not yet been found. At present, most developing strategies against PD are focused either on identifying small molecules, peptides, and peptidomimetics that could directly target α-Syn and its aggregation pathway [1], or on indirect approaches such as the stimulation of intracellular clearance with autophagy promoters [2], degradation pathways based on immunotherapy [3], receptor blocking strategies to inhibit α-Syn spread, or gene silencing [4].

Here we review the current advances in α-Syn-centric treatments for this devastating disease, focusing on those related to α-Syn aggregation inhibition. Therapeutic development requires a deep understanding of the structural properties of α-Syn monomers, oligomers, and fibrils in vitro and in vivo. Revealing the structure/function relationship of native and mutated α-Syn is essential to better comprehend its neurotoxicity. 

## 2. Parkinson’s Disease

Parkinson’s disease is a progressive neurodegenerative disorder that mostly affects the elderly [5,6]. More than ten million people worldwide live with PD, and this number is expected to rise along with increased life expectancy (https://www.parkinson.org/Understanding-Parkinsons/Statistics, accessed on 14 April 2022). The Global Burden of Disease (GBD) reported that PD caused 60,160 deaths and affected 1.4 million people in Europe in 2016 [7]. Today, more than one million Europeans have been diagnosed with PD [8]. This number is forecast to double by 2030 [9]. The number of confirmed PD cases doubled from the last decade of the twentieth century to 2016, with widespread unequal distribution at the world level, which indicates its multifactorial profile [7,10]. 

PD includes various motor (rigidity, slow imprecise movements—bradykinesia, difficulty walking, and tremors at rest) and nonmotor symptoms (gastrointestinal and neuropsychiatric). Gastrointestinal (GIT) dysfunction (obstipation, dysphagia, bloating, and reduced peristalsis) was documented in over 80% of patients and can precede the onset of motor symptoms in PD patients by decades [11,12,13]. Neuropsychiatric symptoms (anosmia, sleep disorders, cognitive defects, autonomic dysfunction, hallucinations, and depression) occur at later disease stages [14,15,16,17]. 

The economic impact of the disease is huge. Both the direct (consultations, medication, and hospitalisation) and the indirect costs (reduced working hours and institutionalisation) are serious causes for concern. The annual European cost is estimated at EUR 13.9 billion, while in the US the total economic burden for 2017 was USD 51.9 billion and will tend to grow as the population ages [18]. The World Health Organisation announced that PD, together with other neurodegenerative disorders, will surpass cancer as the most common group of severe medical conditions by 2040 [19].

Familial PD is genetically inherited in either an autosomal dominant or recessive manner, while sporadic (idiopathic) PD is dependent on gene–environment interactions [8]. The major cause of PD is dopamine deficiency in the striatum, induced by degeneration of neurons originating from *substantia nigra pars compacta*, located in the basal ganglia [20]. Basal ganglia are a key brain hub for movement coordination and muscle contraction control, which explains the origin of PD motor symptoms. Similar to a broad range of other neurodegenerative disorders, PD shares an unclear physiopathology, tightly associated with abnormal protein/peptide aggregation into insoluble, amyloid fibrils [6]. Intracellular protein aggregates mostly composed of α-Syn found in Lewy bodies (LBs) or enlarged neurites (Lewy neurites, LNs) within dopaminergic neurons in the *substantia nigra* are PD’s pathological hallmark [16,21,22]. However, α-Syn aggregates have also been attributed to Alzheimer’s disease [23]. In addition, α-Syn aggregates were related to numerous neurodegenerative disorders, collectively termed “synucleinopathies”, such as dementia with LBs, multiple system atrophy, pantothenate kinase-associated neurodegeneration, frontotemporal dementia, diffuse LB disease, amyotrophic lateral sclerosis (ALS), Parkinsonism dementia complex of Guam, pure autonomic failure, progressive supranuclear palsy, corticobasal degeneration, and Krabbe disease (reviewed in [24,25]).

### 2.1. Genetic Factors Triggering PD

The first evidence of the α-Syn/PD relationship was reported in 1997 when Polymeropoulos et al. [26] demonstrated point mutation in the *SNCA* gene (also known as *PARK1*) encoding α-Syn in a large Italian and three Greek families (that are not genetically related). In the same year, this protein was detected as the main LB constituent and factor in neuron degeneration processes resulting in LN formation [21].

To date, seven missense mutations in the α-Syn-encoding gene *SNCA* (A30P, E46K, H50Q, G51D, A53V, A53T, and A53E) have been linked with autosomal dominant PD [24]. In addition, polymorphisms in the promoter region and a distal enhancer element of the *SNCA* gene affect α-Syn translation and increase PD development risk [27,28]. Moreover, gene locus multiplications, duplications, or triplications have been confirmed to have an important role in the context of inheritance type, symptom development onset, and severity [29,30,31,32]. Besides, mutations in genes encoding leucine-rich repeat serine/threonine kinase 2, LRRK2; vascular protein sorting 35, VPS35; ubiquitin carboxyl-terminal hydrolase isozyme L1, UCHL1; glucocerebrosidase, GBA; parkin RBR E3 ubiquitin-protein ligase, PARK2; phosphatase and tensing homolog (PTEN)-induced kinase 1, PINK1; Parkinson protein 7, PARK7; and Daisuke-Junko-1 protein, DJ1 are involved in the development of autosomal and recessive inheritance of PD [6,33]. 

### 2.2. Environmental Factors Triggering PD

Familial PD cases account for about 5–10% of subjects, while most of the cases are sporadic and more likely caused by interactions of environmental and genetic factors that remain poorly defined [16,34]. Reduced epigenetic *SNCA* silencing has been found in the brains of patients with sporadic PD [35]. Numerous single-nucleotide polymorphisms in *SNCA* have been identified as PD susceptibility variants in several independent genome-wide association studies and meta-analyses, as reviewed in Grosso Jasutkar et al. [25]. Thereby, the regulatory *SNCA* gene regions participate in the development of sporadic PD.

Serious brain injuries, cancer history (melanoma), hormonal changes (postmenopausal hormone distribution), and some autoimmune diseases have been associated with increased PD incidences [36]. Demographic factors including age, gender, and ethnicity may also impact PD susceptibility. PD affects 0.5–1% of the population between 65–69 years, increasing to 1–3% of people over 80 years [14,37]. In addition, PD incidence among men is higher than in women. White Western populations show higher PD prevalence than Asian and Black nations [8]. This can be explained by the higher levels of melanin and neuromelanin in the *substantia nigra*, which may have a neuroprotective effect, but also by socioeconomic state and industrialization level. Environmental risk factors, especially early-life exposure to persistent organic pollutants (POPs), namely organochlorine pesticides, that increase PD prevalence [34,38,39,40]. The link between pesticide toxicity (e.g., dieldrin) and cellular pathology processes in PD (α-Syn aggregation, damage, and death of dopaminergic neurons) has been confirmed [39,41,42]. For example, two decades ago it was shown that accelerated mitochondrial reactive oxygen species (ROS) production caused by rotenone and paraquat led to α-Syn overexpression (more likely for ROS scavenging) that resulted in α-Syn aggregation [43,44]. Besides induction of oxidative modification of monomers, pesticides can stabilise the amyloidogenic partially folded α-Syn conformation [45]. Thus, cellular damage caused by oxidative stress induced by pesticide exposure in combination with genetic background increases the risk for PD [42,46]. Moreover, early-life exposure to pesticides induces a stronger toxic effect than repeated exposure as an adult [34].

In addition to organochlorine compounds, some recent studies connect air pollution, heavy metals, and other pollutants (e.g., 1-methyl-4-phenyl-1,2,3,6-tetrahydropyridine) with PD onset. Chronic exposure of young people to traffic air pollution was positively correlated with symptom appearance [47]. Postmortem analysis of brain tissue samples from young people who lived in contaminated areas revealed accumulated α-Syn deposits [48]. The same author further indicated a connection between PD and exposure to NO_2_ originating from burning fuels [49]. However, the opposite results and a weak connection between air pollution and PD development were also reported [50]. In addition, the increased PD incidences might be associated with the consumption of illicit drugs, such as amphetamine-like stimulants and heroin [40]. 

Numerous early epidemiological studies correlated exposure to heavy metals (Hg, Pb, Cu, Mn, Al, Fe, Mg, Zn, Bi, and Tl) as well as their synergistic and cumulative effect on PD development (reviewed in Bjorkund et al. [51] and Breydo et al. [52]). While metal ions can cause brain damage directly, their effect on PD is based on ROS-inducing effects and direct influence on α-Syn aggregation [53]. Notorious hydroxyl radicals can be produced from hydrogen peroxide in the presence of redox-active metals in trace amounts, such as Fe^2+^ and Cu^+^ via the Fenton’s and Haber–Weiss reactions [54]. Generated ROS can directly or indirectly cause α-Syn misfolding due to oxidative modifications of amino acid side chains. Some metal ions such as Cu^2+^ can directly bind to and promote α-Syn aggregation [55]. Besides, exposure to high metal concentrations was correlated with DNA methylation, Ca^2+^ homeostasis, mitochondrial dysfunction, dopamine synthesis impairment, etc. [56]. Copper can also bind to cysteine residues of enzymes and receptors (D2 dopamine receptors) and alter their activity [54]. In addition, occupational long-term exposure to copper, iron, lead, manganese, etc., alone or in combination, has been associated with an enhanced risk of PD development [57]. Although mercury is known as a neurotoxin, researchers are still conflicted regarding its participation in PD pathogenesis [8]. On the other hand, Wei et al. [56] indicated that some metals could have both neuroprotective and neurotoxic effects depending on concentration and individual metabolic profile (in the case of Cu and Ce) and redox state (in the case of Fe^2+^). 

Besides the discussed environmental risk factors, certain lifestyles and habits are positively correlated with this neurodegenerative disease [58]. Cigarette smoking, consumption of coffee and beverages containing high levels of antioxidants, such as black and green tea, non-steroidal anti-inflammatory (e.g., ibuprofen) drug uptake, physical activity, and a diet based on fruits, vegetables, and fish have a protective action against PD [36]. Phytocannabinoids, which interact with the endocannabinoid system, may play a neuroprotective role in PD [40]. 

Although most PD cases have an unknown etiology, is it certain that the interplay between genetics, previous medical anamnesis, and environmental factors increases PD incidence [35,37,40].

## 3. Alpha-Synuclein

### 3.1. Structure and Function of Native α-Syn Monomer

Much evidence suggests that the chief PD pathological markers are α-Syn oligomers and amyloid fibrils, which are also found in other synucleinopathies linked with LBs [25,59]. Therefore, we will first consider its structural and biophysical properties, which are likely to be a key to its normal and abnormal function. 

**Figure 1:**
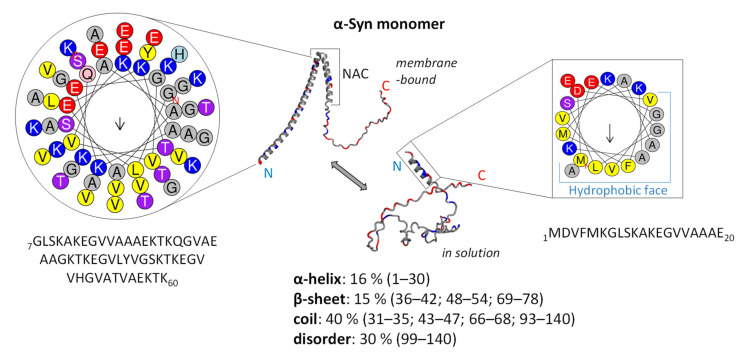
Structural diversity of α-Syn. Helical conformation of α-Syn bound to a membrane and intrinsically disordered α-Syn in solution are presented in the middle. Positively charged amino acid residues are given in blue, and negative ones in red. Secondary structure propensity was obtained by predictor FELLS [63]. Helical projections of N-terminal α-helices (a total of 60 amino acid residues and the first twenty) are generated using the HeliQuest web server [64]. The arrow shows the helical hydrophobic moment. The protein structure was predicted using AlfaFold2 [65]. The 3D protein structure was visualised in PyMOL v2 (available at: https://pymol.org/2/; accessed on 16 April 2022).

The protein α-Syn is small, consisting of 140 amino acids, and lacks both cysteine and tryptophan residues. It is a relatively polar protein (Gravy index = −0.403) composed of 29% charged residues and pI = 4.67. The N-terminus of α-Syn is positively charged and rich in lysine residues (Gravy index for residues 1–60 = −1.883). It is composed of 11-mer imperfect repeats with an XKTKEGVXXXX consensus sequence that can adopt the amphipathic α-helical structure and interact with lipid membranes and vesicles (Figure 1) [60,61]. A central hydrophobic, non-amyloid component (NAC) region (residues 61–95; Gravy index: +0.726), particularly residues 71–82, exhibits a propensity for folding into β-sheets, which is essential for the misfolding and aggregation into fibrils [6]. The highly acidic and proline-rich C-terminus of α-Syn (residues 96–140; Gravy = −1.567) contains a highly flexible intrinsically disordered (ID) region (Figure 1) [62]. 

A common trait of ID proteins (IDPs) is the lack of stable, well-defined 3D structure in the absence of a partner or a ligand under physiological pH and ionic strength [66,67]. Related to that, it is not surprising that α-Syn adopts a monomeric, random coil conformation in an aqueous medium, while its interaction with lipid membranes drives the transition of the protein part (N-terminus and NAC region) into defined α-helical structures (Figure 1) [60,62]. The dynamic C-terminal part is involved in interactions with ligands (ions, polyanions, and polycations), protein partners, and membrane binding [24,68]. Within the C-terminal domain, residues 120–130 were shown to interact with residues 105–115, while positively charged N-terminal residues can interact with negatively charged C-terminal residues, forming a closed α-Syn conformational state [69]. The existence of these long-range interactions was suggested to inhibit spontaneous α-Syn oligomerization and aggregation. On the other hand, lowering the pH (pH 3.0) and increasing buffer temperature induces the folding of unstructured α-Syn into a more ordered secondary structure [52]. 

The α-Syn protein normally exists in a dynamic equilibrium of the disordered monomer and statistically disfavoured helical oligomers, i.e., tetramers [70]. Membrane-bound α-Syn adopts two extended surface-bound α-helices separated by a non-helical linker (Figure 1) [61]. An extended α-Syn helical form can form homotetramers that might be more resistant to aggregation [71,72]. Moreover, these tetramers may participate in a normal function of α-Syn in vesicle trafficking [73]. Nevertheless, the α-helical form is not considered pathogenic [70].

Although it was discovered 30 years ago, the precise physiological function of native, monomeric α-Syn is still incompletely revealed. It is located in the brain, mostly in neuronal presynaptic terminals, bound to synaptic vesicle membranes [74]. In addition, α-Syn was detected in the nucleus, endoplasmic reticulum (ER), Golgi apparatus, and endolysosomal system of neurons [75,76,77,78,79,80]. 

The proposed function of α-Syn in neuron-to-neuron interaction is related to presynaptic homeostasis and neurotransmitter exocytosis on axon terminals [80,81]. It was shown that α-Syn has a role in synaptic vesicle recycling [82,83,84] via inhibition of their release and alteration of N-ethylmaleimide-sensitive factor attachment protein receptor (SNARE) complex-mediated lipid membrane fusion [85]. On the other hand, α-Syn oligomers (tetramers, octamers) facilitate SNARE complex formation and vesicle fusion under physiological conditions [86]. This indicates a potential α-Syn role in modulating dopamine release [6]. Moreover, α-Syn can interact with synaptic proteins, such as phospholipase D2 [87], synphilin-1 [52], synaptobrevin-2 [88], and Rab small GTPases [89]. Furthermore, α-Syn may be involved in synaptic plasticity and vesicle trafficking, brain lipid metabolism, membrane remodelling, membrane channel formation, and modification of their activity [90]. 

Besides the central nervous system, α-Syn has been found in hematopoietic lineages, and low levels of the relevant transcript have been reported in other tissues and fluids (bloodstream, cerebrospinal fluid, CSF) [91,92,93]. Besides the central nervous system, α-Syn is a normal constituent of the enteric nervous system, participating in synaptic vesicle trafficking and exocytosis [94]. However, α-Syn’s physiological role in healthy people’s enteric nervous system is not yet clear [95]. In addition, under specific conditions, α-Syn can even bind to DNA and directly regulate gene expression [77,96].

### 3.2. Neurotoxicity of α-Syn

**Figure 2:**
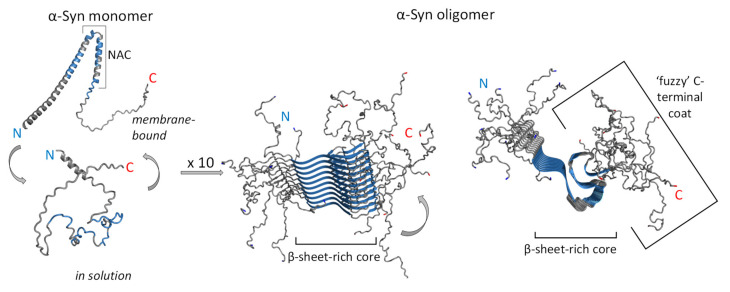
Three-dimensional models of α-Syn monomers (membrane-bound and unstructured in an aqueous solution) and oligomer (PDB ID: 2N0A; frontal and side view). Amino acid residues involved in the intermolecular interactions in oligomers, forming β-sheet rich core are shown in blue. N-terminal regions of monomers are denoted in blue, while C-termini are denoted in red. The monomer structures were predicted using AlfaFold2 [65]. The 3D protein structure was visualised in PyMOL v2 (available at: https://pymol.org/2/; accessed on 16 April 2022).

The central position of α-Syn in PD inheritance, development, and progression has been confirmed by numerous studies [16,25,34,80]. α-Syn presents a spectrum of various species, including monomers (differing in their posttranslational modifications and eventual point mutations), oligomers (exhibiting great variety and heterogeneity), and fibrils (several polymorphs), exhibiting different neurotoxic properties [97]. Analysis of LBs in PD subjects revealed the presence of α-Syn amyloid fibrils rich in β-sheets (Figure 2) [98,99]. Besides the brain, cytotoxic α-Syn oligomers have been found in other tissues and fluids of PD patients [100,101].

It is clear that α-Syn toxicity is connected with its structure, abundance, and aggregation propensity, although the precise relationship between structure and toxicity remains debatable [97]. The α-Syn aggregation propensity depends on specific cellular microenvironment conditions (pH, temperature, ionic strength), macromolecular crowding, and the presence of other ligands/partners (amyloidogenic proteins, metal ions, intermediary toxic species, membranes, and specific lipid molecules) [90,102]. In addition, PTMs induce changes in α-Syn size, charge, structure, and/or conformation, modulating its membrane binding, aggregation, and toxicity [25]. More than 300 different PTMs of α-Syn, including proteolysis, phosphorylation, nitration, glycosylation, *O*-GlcNAcylation, lipidation, oxidation, acylation, ubiquitination, SUMOylation, and N-terminal acetylation, have been described [52,103,104]. The α-Syn C-terminal ID region contains one serine (129) and three tyrosine residues (125, 133, and 135) that can be phosphorylated, altering its toxicity [28]. Surprisingly, 90% of α-Syn found in LBs contain phosphorylated serine 129 [105], suggesting the necessity of this modification in LB formation [106]. On the other hand, phosphorylation at tyrosine 125 does not affect α-Syn fibrillization [107], while the effect of phosphorylation at tyrosine 133 and tyrosine 135 on α-Syn remains unknown [108]. On the other hand, phosphorylation of tyrosine 39 at α-Syn’s N-terminus provokes its aggregation and directly impairs the α-Syn interactions with chaperones, increasing its neurotoxicity [109]. Phosphorylated α-Syn species present target proteins for ubiquitination in various synucleinopathies [108]. Besides phosphorylation, 10 to 30% of α-Syn in LBs is truncated in the N- or C-termini [23]. In addition, markedly elevated SUMOylated α-Syn (conjugates of small ubiquitin-like modifier, SUMO and lysine residues) was found in LBs [110]. Several comprehensive in vitro studies reported site-specific effects of SUMOylation on α-Syn aggregation propensity [108]. SUMOylation promotes α-Syn aggregation directly and by blockage its degradation [110], whereas SUMOylation of other lysine residues inhibits the α-Syn aggregation pathway [111,112]. Alternatively, O-GlcNAcylation of several threonine residues found in vivo in mice and humans does not increase α-Syn toxicity [108]. Moreover, several specifically synthesised and enzymatically O-GlcNAcylated threonine residues inhibit full-length α-Syn fibrillization [108,111], while some reduce the toxicity of extracellular α-Syn fibrils involved in PD’s spreading [113]. Besides the mentioned PTMs, non-enzymatic oxidative modification (e.g., methionine oxidation, nitration, and oxidative tyrosine dimerization) and modification by oxidative dopamine adducts impact α-Syn’s aggregation propensity [52]. Both nitration and oxidation of α-Syn have been suggested as possible mechanisms responsible for dimerisation and oligomerisation through dityrosine crosslinking [104]. In α-Syn isolated from human LBs four tyrosine residues were found nitrated [103]. 

Aggregation of α-Syn in vitro shows a sigmoidal profile, indicating a nucleation-polymerization mechanism. This means that soluble α-Syn monomers form intermediate, transient oligomeric structures and then assemble into more structurally ordered insoluble fibrillar aggregates. In addition, α-Syn monomers follow a nucleation process by further monomer addition to form β-sheet-rich amyloid fibrils [90]. In this process, a rate-limiting step is the spontaneous formation of small metastable oligomeric intermediates, also known as fibril nuclei [52]. Therefore, α-Syn aggregation is highly cooperative, and even a small amount of modified/misfolded α-Syn could have a large impact on kinetics and aggregate dispersal.

The α-Syn protein builds fibrils by converting either all or part of the previously unstructured polypeptide into well-defined, β-sheet-rich secondary structures (Figure 2). In accord, α-Syn oligomers exhibited high structural diversity, i.e., some are β-sheet-rich (Figure 2), while others are disordered [114]. As mentioned earlier, seven point mutations—A30P, E46K, H50Q, G51D, and A53E/T/V—found in PD patients provoke α-Syn aggregation and further accumulation, suggesting the importance of these regions in α-Syn misfolding. Although detailed structural analyses revealed that the PD-related point mutations do not influence the α-Syn 3D structure, A30P decreases α-helical propensity, while E46K mutation enhanced the contacts between N- and C-termini [52]. Finally, the A30P mutation favours α-Syn oligomerisation, while A53T and E46K mutations promote fibrillation.

In the last decade, advanced crystallography, cryoelectron microscopy (cryo-EM), and nuclear magnetic resonance (NMR) techniques revealed that residues 45–57 of α-Syn are key for the β-strand/β-strand interactions required for toxic fibril formation [6]. By using solid-state NMR (ssNMR), Madine et al. [115] identified residues _77_VAQKTV_82_ as the key region for α-Syn self-aggregation. In addition, Mirecka et al. [116] showed that residues α-Syn _37_VLYVGSK_43_ (β 1) and _48_VVHGVAT_54_ (β2) adopt a β-hairpin connected by a β-turn formed by _44_TKEG_47_ residues (Figure 2). In addition, the high-quality cryo-EM density map showed that the core of the acetylated wildtype α-Syn fibril consists of residues 37–99, while N- and C-termini remain flexible [117]. Nevertheless, the NAC-region residues form a rigid β-sheet core with solvent-exposed hydrophobic clusters, while the flexible N- and C-terminal solvent-accessible regions form a dynamic ‘fuzzy coat’ around the fibril core [100,118,119]. The fibrillar β-sheet arrangement adopts a serpentine Greek key topology stabilised by electrostatic and additional interactions between the NAC region and the N-terminus [117,120]. The already mentioned phosphorylated tyrosine 39 is in the centre of the fibril core and forms an electrostatic interaction network with eight charged residues in the α-Syn N-terminal region [121]. Finally, four distinct types of full-length α-Syn fibrils were identified by cryo-EM: type 1a ‘rod’, type 1b ‘twister’, type 2a, and type 2b polymorphs [122,123,124]. Taken together, all α-Syn fibril polymorphs consist of several protofilaments containing a cross-β structure in which surface-exposed β-strands are arranged in a parallel pattern (Figure 2). 

Besides the NAC region, flexible N- and C-termini are shown to be actively involved in the α-Sin aggregation mechanism [125]. A recent NMR study emphasised the role of the N-terminal region in fibril elongation [119]. The first 11 N-terminal acetylated residues on the α-Sin monomer interact with ID regions (C-terminal tails) of preformed acetylated fibrils. 

The exact function of α-Syn in PD etiology, as well as the molecular mechanism that underlies dopaminergic neuron degeneration, is not completely comprehended. It is unknown which species of the α-Syn aggregation pathway (including which fibril polymorph(s)) is/are causing neuronal death. More importantly, the mechanism of how α-Syn aggregates induce neuronal death is yet not clear [126]. The cellular pathological consequences of these processes are related to oxidative stress, mitochondrial dysfunction, impairment of ER–Golgi and synaptic vesicle trafficking, membrane integrity loss, alteration of the ubiquitin–proteasome system, impairment of the immune system associated with inflammation, cell aging, epigenetic changes, and synaptic dysfunction [16,24,25,80,127]. The abnormal accumulation of nuclear α-Syn has been connected to DNA damage and neurotoxicity [128,129]. 

The putative neurotoxicity caused by α-Syn oligomers and aggregates has been correlated with impaired membrane permeability and their cell-to-cell transfer [22,97,130]. Fusco et al. [100] suggested that α-Syn oligomers can strongly interact with membranes through a highly hydrophobic region at the surface and via a rigid core rich in β-sheets that can be inserted into the lipid bilayer and perturb membrane integrity in vitro. Increased membrane permeability leads to enhanced ROS accumulation and increased levels of basal intracellular Ca^2+^, causing cell death [131,132]. On the other hand, A30P and G51D missense mutations, found in familial PD cases, exhibited lower membrane affinity and might be toxic in the form of soluble oligomers [133]. Pertinent to that, unfolded α-Syn monomers can bend and stabilise vesicle membranes in vitro [134], which may in turn inhibit membrane fusion events during exocytosis [73]. Mutations that abrogate tetramer formations become insoluble, highly phosphorylated, C-terminally truncated proteins and further accumulate within lipid vesicles [70]. Higher accessibility of C-terminal tails of α-Syn fibrils is responsible for binding to the proteasome and blocking proteolytic activity [135].

Cell-to-cell transfer mediated by receptors, or the so-called ‘prion-like’ hypothesis, suggests that α-Syn oligomers can migrate between neurons [6]. Moreover, the acidic α-Syn C-terminus interacts with alkaline patches on the cell surface receptors, such as lymphocyte activation gene 3 (LAG3) and amyloid precursor-like protein 1 [68]. Amyloid fibril formation dramatically enhances (>200 times) binding to receptors due to the more accessible C-terminus that is shielded in the monomer. Moreover, serine 129 phosphorylation additionally strengthens the electrostatic interactions between α-Syn and the receptors, accelerating the PD-like pathology in mice [68]. It has also been shown that heparan sulphate proteoglycans on the cell surfaces participate in the uptake of α-Syn amyloid fibrils through endocytosis [136]. In addition, microglial α-Syn uptake is species-specific. For example, only β-sheet-enriched oligomers can be internalized via Toll-like receptor 2 (TLR2) found in neurons and glial cells [137]. In this way, oligomers spread and propagate LB formation throughout the *substantia nigra* and into extranigral regions. Propagation of α-Syn in mice following injection of toxic α-Syn forms into the brain has been confirmed (reviewed in [25]). In humans, evidence of cell-to-cell transfer of toxic α-Syn species is still scarce.

Extracellular α-Syn oligomers can exhibit neurotoxic and neuroinflammatory activity [138]. Immune system impairment in PD is connected to α-Syn overaccumulation in microglia, which leads to stronger activation of adaptive immunity [139]. Namely, abnormal α-Syn recognized as an autoimmune antigen leads to T cell activation, resulting in inflammation and triggering neurotoxic cellular pathways, leading to faster disease progression [140]. The interaction of neuron-released α-Syn uptaken by the glial cells results in cellular stress that can induce inflammatory responses as well [141,142]. 

Neurotoxicity and neuroinflammation processes related to the central nervous system have been extensively studied, while data related to the enteric nervous system are scarce. Native or phosphorylated α-Syn forms, as well as proteinase-K-resistant α-Syn aggregates, have been observed in intestinal samples of PD patients [143]. At the moment, the toxicity mechanism of α-Syn species in the GIT is not clear, but recent studies indicated a positive correlation between intestinal inflammation and increased α-Syn expression and accumulation [144,145]. Moreover, monomeric and oligomeric α-Syn species can act as chemoattractants for immune cells [144]. Increasing evidence indicates that α-Syn pathology could start in the enteric nervous system and then spread to the brain, inducing pathogenesis and progression of PD and related disorders [91]. In addition, α-Syn toxicity spreading via the vagal nerve has been shown in animal models [146,147]. Therefore, PD pathology can propagate between GIT and the brain in both directions [148,149]. Revealing the interaction of GIT dysfunction with α-Syn accumulation, aggregation, and spreading is crucial for understanding PD etiopathogenesis [13]. 

Taken together, PD and other synucleinopathies’ pathogeneses are influenced not only by α-Syn oligomerisation and aggregation, but also by numerous factors, including genetic predisposition, toxic insults, chaperone system failure, proteasomal misfunction, rare, early-onset cases of autosomal origin, and oxidative damage [52].

## 4. Therapies and Strategies against PD Related to α-Syn

Effective strategies for preventing or slowing down α-Syn aggregation and neurotoxicity are urgently needed to address the exponential increase in PD. Early detection is a key to its prevention and treatment. Currently, PD diagnosis is based on the recognition of clinical symptoms via medical examination and anamnesis, at which point neurodegeneration is already advanced. Dopamine transporter single-photon emission computed tomography helps confirm the diagnosis [15]. In the last years, new diagnostic tools for early diagnosis of synucleinopathies, with a special interest in PD, have emerged. The diagnostic value of α-Syn as a PD biomarker has been broadly studied since its aggregation is an early event that precedes the onset of clinical symptoms [150]. Although recently developed, protein-misfolding cyclic amplification (PMCA) and real-time quaking-induced conversion (RT-QuIC) based on the amplification of misfolded proteins present in biological fluids or tissue samples are ultrasensitive; only a few studies have been performed to test their accuracy as diagnostic tools [90]. Considering prodromal GIT symptoms and the occurrence of toxic α-Syn species, GIT samples containing enteric neurons might be a valuable source in PD diagnostics and monitoring its progression from clinical and biochemical aspects [151]. On the other hand, the implementation of genetic testing in PD diagnosis is at the research and/or clinical levels at the moment. Reasons for this are high expense, ethical concerns, and, most importantly, the complex profile of PD progress, requiring comprehensive result interpretation. The genetic approach to diagnosis has a bright future, taking into account novel data related to spinal muscular atrophy and familial amyloidosis [152]. Further development of early-stage diagnosis tools is still needed to standardize operating procedures and would certainly require the strengthening of the partnership between researchers and clinicians.

### 4.1. Current PD Treatments

Present approaches in PD therapy include motor and nonmotor symptom treatment. No standardised therapy addressing the disease causes and primary development and slowing its progression is available. One of the biggest challenges in efficient PD therapy development is the lack of reliable and sensitive disease progression biomarkers [17,150]. 

Neurological PD symptoms, depression, and anxiety are treated by selective serotonin reuptake inhibitors, while dementia is treated with cholinesterase inhibitors [15]. Motor dysfunction in PD is managed with symptomatic therapy, such as dopamine precursor levodopa for dopamine replacement. L-3,4-dihydroxyphenylalanine (L-DOPA) can cross the blood–brain barrier (BBB) and is converted into dopamine through decarboxylation in pre-synaptic terminals [153]. Dopamine-mimicking medications (pramipexole, apomorphine, or ropinirole), as well as inhibitors of enzymes involved in dopamine catabolism (catechol-*O*-methyltransferase and monoamine oxidase type B), are presently used [154,155]. The application of levodopa–carbidopa intestinal gel or enteral suspension was officially introduced as a therapy for advanced PD stages in 2004 in the EU and in 2015 in the USA [156,157]. 

As the disease progresses, treatment resistance and side effects emerge, and non-motor symptoms worsen. Continual application of dopamine-related PD drugs is linked with a wearing-off phenomenon (end-of-dose failure), dyskinesia, and other negative side effects on mental health [158].

Glucagon-like peptide-1 receptor (GLP-1R) agonists, commonly used in Type 2 diabetes mellitus treatment, have recently been considered potential candidates for PD treatment [58]. Reported studies confirmed their neuroprotective and anti-inflammatory activity [159,160]. This approach targets chronic inflammation in microglia and stimulates immune modulators. Proposed GLP-1R agonists mobilise cellular protective mechanisms, including neurotrophic factor production and recovery of brain insulin sensitivity [160]. Neurotrophic factors are important for the development and homeostasis of the nervous system, as well as for regeneration and remyelination processes. However, this approach is still not adapted and accepted as a regular PD treatment. 

Besides medication therapy, several surgical procedures have been developed. Deep brain stimulation is a treatment recommended for patients with inadequate response to drugs, while focused ultrasound is a treatment intended for patients with tremors as the main motor symptom and gait pattern issues [58]. However, this procedure enhances the risk of infections and only offers transient motor symptom relief. Additionally, extensive research in the field of cell transplantation and gene therapy in PD treatment is ongoing [161,162,163,164]. 

To overcome the existing treatment limitations related to PD onset and modulation of its progression, novel treatment approaches mostly target the underlying α-Syn aggregation mechanisms [165]. Monomers, oligomers, and α-Syn fibrils are one of the leading and the most persuasive targets for PD modification therapy today [59]. Intrinsically disordered α-Syn is an undruggable target and a real challenge for the design of therapeutics based on molecular recognition. To date, several approaches targeting the α-Syn aggregation pathway and proteostasis mechanisms are at different levels of clinical trials.

### 4.2. α-Syn-Targeted Therapies

**Figure 3:**
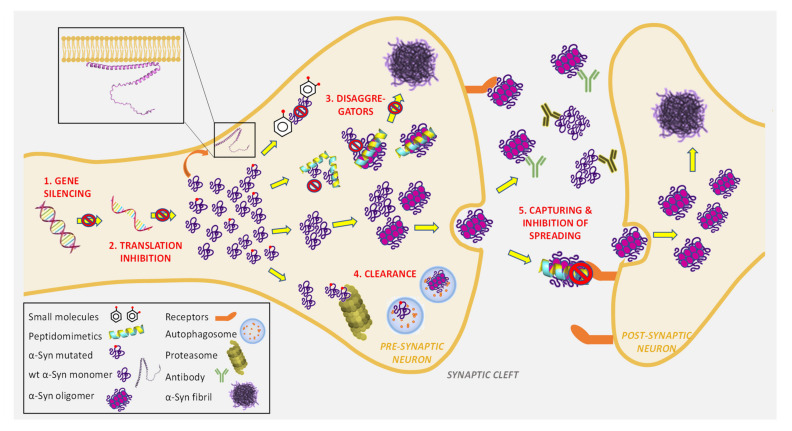
Five major developing α-Syn-targeting therapeutic strategies. They include reducing α-Syn transcription (**1**) and translation (**2**), inhibiting α-Syn aggregation by disaggregators involving low-molecular-weight compounds identified by high-throughput screening and rationally designed peptides or peptidomimetics (**3**), enhancing α-Syn clearance and degradation through autophagy and the ubiquitin-proteasome system (**4**), and capturing the toxic α-Syn forms in the extracellular space and blocking their transcellular spreading via immunotherapies (**5**).

Looking for the first disease-modifying therapy for PD, researchers and pharmaceutical companies are developing α-Syn-targeted agents in proof-of-concept clinical trials [59]. Potential strategies related to α-Syn involve a wide range of actions: transcription and translation blockage, inhibition of aggregation plus degradation of formed oligomers, stimulation of intracellular clearance with autophagy promoters, and the inhibition of the toxic form spreading, mostly based on immunotherapy (Figure 3) [2,3,25,58,158]. In addition, the propensity for α-Syn aggregation can be decreased by preventing some PTMs that promote its self-assembly. For example, specific SUMOylation blockers might be used as therapeutics to prevent intracellular α-Syn aggregation [110]. However, it should be emphasised that α-Syn can have multiple different PTMs concurrently in vivo, while the majority of the related studies investigate a single PTM individually [108]. The fact that α-Syn is IDP and that it constantly changes the structure in time makes the strategies based on molecular recognition more difficult. Besides acting on α-Syn aggregation directly, an interesting approach targeting lipid in the presynaptic plasma membrane regions has also been proposed. Glycosphingolipid glucosylceramide can interact with α-Syn and promote its aggregation. Related to that, the application of brain-penetrant glucosylceramide synthase inhibitors reduces α-Syn aggregate accumulation and improves cognitive function in the α-Syn-mutant mouse PD model [36].

#### 4.2.1. Reduction of α-Syn Expression and Synthesis

Since *SNCA* gene locus multiplication is one of the dominantly inherited PD causes, reducing α-Syn expression is expected to have therapeutic value (Figure 3). *SNCA* expression is regulated by a noncoding distal enhancer element [166]. Expression control by far regions can include complex chromatin loops and epigenetic modifications. Small molecules, proteins, RNA derivates, oligonucleotides, β2-adrenergic receptor (β2AR) agonists, and ribozymes are selected as potential candidates to decrease α-Syn accumulation [25,167].

Among proposed solutions that target transcription, one of the most extensively studied is a group of BBB-penetrant β2AR agonists [168,169]. β2AR agonists are commonly applied in asthma treatment. The present approach is based on *SNCA* expression regulation by β2AR ligands through histone 3 acetylation of *SNCA* promoters and enhancers. However, the reported data on the effect of β2AR agonists on *SNCA* expression is still not uniform in the context of the desirable effect [170,171].

To downregulate the *SNCA* gene, ongoing studies rely on small interfering RNA (siRNA), short hairpin RNA (shRNA), microRNA (miRNA), and antisense oligonucleotides (ASOs) [165]. MicroRNA and ribozymes downregulated α-Syn expression in cell cultures and rats, respectively, but the updated outputs are not available [25,172,173]. Several studies on animal models, cell cultures (i.e., cultured human neuroblastoma SH-SY5Y cells), and primary neurons related to siRNA, shRNA, and ASOs showed promising results related to α-Syn expression reduction without negative effects on dopamine metabolism and immune response [174,175,176]. ASO application in *SNCA* downregulation is inspired by previous data on other neurodegenerative diseases sharing similar pathophysiology (single protein/peptide dysfunction and aggregation) [177,178]. Cole et al. [179] demonstrated that ASOs reduce α-Syn biosynthesis, decrease the number of its aggregates, and preserve dopaminergic function (keeping tyrosine hydroxylase activity) in the central nervous system and CSF of PD patients in a dose-dependent manner. The authors recommended this approach in future drug development as more adequate than immunotherapy since it targets intracellular RNA instead of already misfolded α-Syn in the extracellular space [179].

The described protocols succeeded in reducing α-Syn expression. However, they created long-termed side effects in the experimental animals, resulting in the loss of dopaminergic neurons [180,181]. Given that α-Syn’s physiological role is tightly linked with synaptic functions [82], it is expected that chronic *SNCA* downregulation can have adverse effects. Several studies emphasised the critical range of *SNCA* expression levels required for maintaining dopaminergic neuron function [165]. Indeed, α-Syn suppression initiates a neuronal-mediated inflammatory cascade, involving both the innate and adaptive immune systems, that ultimately results in neuronal death [182]. Thus, further genetic-based therapies should provide a partial reduction of α-Syn biosynthesis and careful regulation of *SNCA* expression to preserve dopaminergic neuron function and avoid neurotoxic side effects. To date, protocols based on silencing *SNCA* in vivo have not been advanced enough to reach clinical studies. Moreover, the challenge of genetic material delivery and crossing the BBB has yet to be overcome (reviewed in Teil et al. [165]). 

To resolve the issue of precise *SNCA* expression regulation, stem cell transplantation in combination with CRISPR/Cas9 technology has been suggested. This approach provided a precise, genetically controlled experimental system for developing an efficient PD treatment [28]. Moreover, patient-induced pluripotent stem cells (pIPSC) present a valuable source for cell replacement therapy, circumventing immune rejection issues [183]. Soldner et al. [28] described a novel strategy to functionally dissect the cis-acting *SNCA* allele-specific regulatory elements (distal enhancers) by combining genome-wide epigenetic information with CRISPR/Cas9-mediated deletion and insertion/exchange in pIPSC. In this way, PD-associated risk allele variants in noncoding distal enhancers were identified. Moreover, brain-specific transcription factors, i.e., EMX2 and NKX6-1, specifically bind to these enhancers in a sequence-dependent manner, finally regulating *SNCA* expression [28]. 

The missense mutation of *SNCA* and *LRRK2* genes were corrected in vitro by another gene-editing approach based on zinc-finger nucleases (ZFNs) [166,184]. Through ZFN genome editing, the reporter gene sequence was introduced in-frame downstream of the *SNCA* gene in order to preserve the native *SNCA* expression level [166]. This ensured full retention of known and unknown upstream and downstream genetic elements controlling *SNCA* expression.

Besides, several small molecules have been recently tested for α-Syn reduction at the translational level [92,185,186]. Two designed molecules were extensively studied: synucleozid and posiphen. Synucleozid targets the α-Syn translation regulatory element iron-responsive element (IRE) structure and inhibits *SNCA* translation in SH-SY5Y cells [68]. At low Fe concentrations, iron regulatory protein (IRP) is bound to IRE, while at high Fe levels, IRP binds Fe, releasing the *SNCA* mRNA to undergo translation [187]. Binding synucleozid to IRE stabilises the IRP/*SNCA* mRNA complex and represses translation [68]. Posiphen increases IRP affinity to IRE, thus preventing the association of *SNCA* mRNA with the ribosome and inhibiting translation [188]. Similarly, posiphen suppresses the translation of amyloid-β precursor protein that is involved in Alzheimer’s disease pathogenesis [189]. Posiphen exhibited a protective effect on motor activity in transgenic mice expressing mutant α-Syn forms, and at the moment is under clinical trial [92].

#### 4.2.2. Direct Inhibition of α-Syn Aggregation by Small Molecules

Given the range of the societal and economic burden caused by PD, enormous efforts have led to an increased number of clinical and preclinical trials regarding α-Syn aggregation inhibition [16,58,88]. At the moment, dozens of potential candidates, including small molecules and peptides that inhibit α-Syn aggregation, are under some level of preclinical or clinical trials [165,190,191,192]. These molecules are also known as disaggregators (Figure 3). Based on their mechanism of action, they can be classified into the following groups: (i) oligomer formation modulators; (ii) inhibitors of amyloid fibril formation; (iii) inhibitors of oligomerisation targeting the NAC region; (iv) molecules displacing α-Syn from the membrane; and (v) compounds that direct misfolded or aggregated α-Syn to the proteasome [25]. Despite numerous studies, in most cases the nature of the interactions between disaggregators and α-Syn has still not been explained properly [126]. 

Phenolics are suitable α-Syn disaggregators (Figure 3), having (i) the aromatic moieties that allow non-covalent interaction with hydrophobic β-sheet-rich regions of α-Syn oligomers, and (ii) hydroxyl groups, responsible for aggregation disturbance and destabilization of oligomer structures [193]. An additional α-Syn anti-aggregation mechanism of polyphenolics is based on the chelation of metal ions that favour the α-Syn aggregation process [194]. Pertinent to this, the α-Syn anti-aggregation potentials of various natural and synthetic polyphenolic compounds have been tested. Naturally occurring tannic, nordihydroguaiaretic, and rosmarinic acids, as well as curcumin and myricetin, altered α-Syn misfolding in vitro and inhibited its oligomerisation and propagation in vivo [195,196,197,198]. Masuda et al. [199] performed an extensive in vitro screening of 79 different low-molecular-weight compounds with diverse chemical structures to select those with α-Syn anti-aggregation properties. They identified several polyphenol compounds, baicalein, delphinidin, dopamine chloride, epigallocatechin gallate, and gallocatechin, that turned out to be potent inhibitors in vitro [199,200]. These compounds induced lower toxicity of α-Syn soluble monomers and oligomers in cell cultures, as well. More precisely, flavone baicalein acts as an α-Syn disaggregator by stabilising the native protein conformation [201].

Derivatives of dietary polyphenols originating from gut microbiota-based metabolism (3-hydroxybenzoic acid, 3,4-dihydroxybenzoic acid, and 3-hydroxyphenylacetic acid) exhibited therapeutic potential. These compounds inhibited α-Syn oligomer formation in vitro and reduced their cytotoxic effect in cell-based systems [202]. In addition, they delayed motor symptom development in other neurodegenerative disorders sharing the similar pathophysiology of proteinaceous inclusion formation. 

Alternatively, some natural steroid compounds have been reported to be useful α-Syn fibril disaggregators. Squalamine, steroid polyamine, and similar metabolites obtained from sharks were originally applied as anticancer and antibacterial agents [203], as well as in obesity treatment and tissue regeneration [204]. Squalamine’s proposed action mechanism relies on the displacement of α-Syn toxic forms at the plasma membrane. Squalamine and its derivatives can inhibit the lipid-induced initiation step in the α-Syn aggregation process [205]. A year later, Perni et al. [206] showed that another related aminosterol, trodusquemine, inhibited the fibril-dependent secondary α-Syn aggregation pathway [206]. Trodusquemine dramatically lowered the number of α-Syn inclusions and consequently abolished muscle paralysis and prolonged the lifespan in the *Caenorhabditis elegans* PD model. Oral application of trodusquemine was beneficial against α-Syn aggregation and paralysis symptoms, while the synthetic squalamine salt ENT-01 is a subject of an ongoing clinical trial [25]. Given their BBB penetrability, the mentioned aminosterols should be considered therapeutic candidates for PD and related synucleinopathies.

Several α-Syn conformational modulators have been proposed to be used as therapeutics [207]. These molecules target specific, conserved α-Syn conformations, acting more selectively. For example, binding polycationic polyamines such as spermine to acidic α-Syn C-terminus accelerates its aggregation [208] through the conformational transition from closed to an open form [69]. As mentioned above, C- and N-termini of monomeric α-Syn can bind via electrostatic interactions and form a closed conformational state [69]. The C-terminal-tail-binding spermine causes a release of the N-terminus, followed by opening of the α-Syn structure. 

Based on systematic high-throughput screening of a large database (20,000 drug-like compounds) and medical chemistry optimisation, promising results were obtained for oligomer modulator anle138b (3-(1,3-benzodioxol-5-yl)-5-(3-bromophenyl)-1H-pyrazole) [209]. Anle138b specifically inhibited α-Syn oligomer formation in vitro and in human cell line HEK293 [200]. It exhibited a protective capacity against rotenone toxicity and motor dysfunction development in A30P α-Syn transgenic mice, slowing disease progression [210]. Further, the first-in-human, placebo-controlled, double-blind, randomized trial assessing anle138b safety, tolerability, and pharmacokinetics in healthy volunteers was conducted in 2020 [165]. Additional anle138b-related clinical studies are ongoing [25]. 

Following a similar screening method, synthetic compound BIOD303 was selected from the Maybridge Ro3 fragment library for its ability to inhibit α-Syn conformational change upon spermine addition [207]. BIOD303 binds specifically to α-Syn monomers, causing conformational change followed by significantly reduced α-Syn aggregation in neurons.

By a recently established fast, high-throughput α-Syn anti-aggregation screening method, Pujols et al. [211] screened 14,400 compounds from the Maybridge HitFinder Collection, seeking putative α-Syn aggregation inhibitors. They identified a small aromatic molecule, SynuClean-D (2-hydroxy-5- nitro-6-(3-nitrophenyl)-4-(trifluoromethyl)nicotinonitril), that targets the NAC region within the α-Syn fibril core (detected by molecular docking and simulations) [190,212]. NMR studies confirmed no interaction between SynuClean-D and monomeric α-Syn. SynuClean-D inhibited wildtype α-Syn aggregation as well as the familiar A30P and H50Q variants in a substoichiometric molar ratio in vitro [190]. Moreover, SynuClean-D inhibited α-Syn aggregation in muscle, resulting in motility recovery in two *C. elegans* PD models, which express α-Syn either in muscle or in dopaminergic neurons. At the same time, based on its potential to disassemble amyloid fibrils, SynuClean-D exhibited protective and nontoxic effects on dopaminergic neurons and in *C. elegans* [211]. Very recently, SynuClean-D has shown disaggregation activity on different α-Syn amyloid polymorphs found in different synucleinopathies and disease progression stages [212]. Furthermore, SynuClean-D’s activity is based on conformation-dependent interactions. Taken together, SynuClean-D has been suggested as a very promising therapeutic for PD and other synucleinopathies. 

The so-called “molecular tweezers” represent the first class of artificial receptor supramolecule designed to host a drug candidate and have had promising results in animal tests. These U-shaped molecules consisted of aromatic side walls, forming a rigid, concave cavity that contains anionic groups that should enable capturing of cationic drug candidates [213]. Regarding the development of PD therapeutics, “molecular tweezers” were designed to inhibit key interactions in α-Syn oligomerisation via interaction with α-Syn lysine residues and disrupting hydrophobic and electrostatic interactions at the monomers’ interface [214,215]. Emerged supramolecule CLR01 disaggregated formed fibrils [216] and inhibited α-Syn aggregation in cell cultures and zebrafish embryos [214]. 

For inhibition-based studies, high-throughput screening has identified several promising α-Syn aggregation inhibitors [211,217]. Being a dynamic target, disordered α-Syn impedes structure-based drug design [67,218]. Therefore, multiple contact points organised over a large surface area are required for the specific binding of other molecules to α-Syn [126]. The additional challenge in finding potential therapeutic molecules that can specifically interact with α-Syn amyloid fibrils (formed from recombinant α-Syn) in vitro is that they conformationally differ from those observed in patients’ brains [219]. Moreover, synthetic α-Syn fibrils formed under given conditions exhibit different seeding activity and neurotoxicity in cells and when inoculated in rat brains [220,221]. Current inhibition-based studies primarily target the rigid α-Syn fibril core. However, most small molecules that directly bind α-Syn also bind various biomolecules and cause numerous side effects. The lack of sensitivity and specificity of the identified candidates does not satisfy the requirements for clinical drugs [100].

#### 4.2.3. Direct Inhibition of α-Syn Aggregation by Short Peptides and Peptidomimetics

The issue related to the lack of selectivity of small molecules that can bind disordered α-Syn monomers and oligomers can be circumvented by peptide-based strategies. Indeed, biomolecules such as peptides, nucleic acids, and oligosaccharides are more suitable for these purposes, providing needed selectivity and affinity. The highly selective and reversible interactions of peptides decrease side effects and toxicity [222]. Pertinent to this, in silico-designed mimetics of α-helices, β-strands, and β-sheets containing surfaces complementary to those of α-Syn monomers, oligomers, or preformed fibrils can be prevent α-Syn aggregation (Figure 3) [223,224]. Rationally designed molecules for the recognition of (i) the α-Syn region responsible for assembly, known as the self-recognition element, (ii) the α-Syn region required for the interactions with membranes, and (iii) specific native α-Syn conformational states have become prevalent as a result.

To block α-Syn oligomerisation, two peptidomimetics that can interfere with the membranes and disable aggregation were designed: NPT100-18a and NPT200-11. Wrasidlo et al. [225] developed NPT100-18a, a cyclic peptidomimetic analogous to the _96_KKDQLGK_102_ sequence of α-Syn that most frequently binds to another α-Syn molecule during oligomerisation. In addition, NPT100-18a disabled interactions between α-Syn and plasma membranes. However, this peptidomimetic showed limited oral bioavailability, relatively poor BBB penetration, and other liabilities that disallowed their advancement as therapeutic candidates [226]. To overcome these limitations, NPT200-11 designed for oral administration (UCB and Neuropore Therapies) reduced α-Syn pathology in the cortex of line 61 transgenic mouse model overexpressing human native α-Syn [226]. NPT200-11 interacts with part of the α-Syn C-terminus, diminishing α-Syn aggregation, neurodegeneration, and neuroinflammation in transgenic mice overexpressing wildtype α-Syn or α-Syn coupled with GFP [59,226]. NPT200-11 successfully completed safety trials in healthy volunteers, and further phase I testing was performed. Fusion protein NPT088 (Proclara Biosciences), derived from an active part of g3p (bacteriophage M13 capsid protein) and human recombinant IgG1-Fc protein, has also been shown to impair α-Syn amyloidal [227]. NPT088 was tested against the amyloid-β protein in Alzheimer’s disease and demonstrated good safety and tolerability, while its pharmacokinetics in PD patients still has to be estimated.

Very recently, Bavinton et al. [126] intended to stabilise the α-Syn α-helical conformation in the presence of a lipid bilayer through the complementary surfaces of rationally designed peptidomimetics. Ten oligobenzamides with the potential to form helix–mimic interactions analogous to that of the helixhelix scaffold were selected and tested for α-Syn anti-aggregation activity. Oligobenzamides targeting E20, Q24, and E28, as well as those targeting E46, H50, and A53 of α-Syn stabilised its monomeric form and inhibited α-Syn aggregation, as demonstrated by the thioflavin-T assay, EM, and circular dichroism in the presence of liposomes/vesicles [126]. 

Finding that the NAC region is mainly involved in α-Syn/α-Syn interactions and further fibrilisation by forming β-hairpins, short peptides that can bind to self-recognition elements in α-Syn monomers were rationally designed. These so-called β-sheet breakers required β-sheet-breaking amino acid substitutions, i.e., the addition of N- and C-terminal blocking groups, replacement of amide bonds with ester linkages, and introduction of R-disubstituted amino acids such as R-aminobutyric acid [115]. In order to disable hydrogen-bond forming on one side, N-methylated backbone amide groups were involved. N-methylated peptides are designed to provide high water solubility, proteolysis resistance, and diffusion through membranes [228,229].

Following these principles and focusing on _77_VAQKTV_82_ involved in forming ordered β-hairpin conformation in the fibrils, Madine et al. [115] synthesized four peptides: mVTGVTA, VTGmVTA, VmAQKTV, and VAQKTmV. Only VAQKTmV was effective in preventing α-Syn from forming large, insoluble fibrillar species in vitro. 

Synthetic heptapeptide RKVmPYT has the potential to inhibit amyloid-β peptide aggregation [230]. Similarly, this peptide could bind to α-Syn as well [231]. To assess how the conformational flexibility of this heptapeptide influences the interactions with disordered α-Syn, partially constrained mPhe was substituted with its D-analogue, D-mPhe, less prone to proteolysis, as well as with the conformationally restricted tetrahydroisoquinolinecarboxylic acid (Tic) and unconstrained Ala and Phe [231]. All peptides could interact with α-Syn, although the highest activity was observed for the more flexible peptide backbone (Ala instead of mPhe), suggesting an important role of the phenylalanine side chain in α-Syn recognition. In addition, the binding of these peptides to α-Syn did not induce any ordered conformation in monomeric α-Syn. Moreover, instead of inhibiting α-Syn aggregation, these peptides accelerated its aggregation [231].

To stabilize partially structured α-Syn monomers, engineered proteins such as β-wrapin AS69, which binds to the α-Syn N-terminus with high affinity, were employed [97,232]. β-Wrapins are proteins that can bind to α-Syn, generated by random mutagenesis in the gene encoding ZAβ3, a homodimer linked via the S–S bridge that can bind to the amyloid-β peptide. The sequestration of a β-hairpin in the α-Syn β1 region (37–54) by binding to AS69 inhibits α-Syn aggregation [116]. The substoichiometric inhibition implies that AS69 does not bind to α-Syn monomers. Instead, it is more likely that a small percentage of AS69 binds to α-Syn oligomers, impeding the following conformational transition to ordered amyloid fibrils, or that AS69 binds with high affinity to fibril ends, abolishing fibril elongation. Moreover, the viability of SH-SY5Y cells was recovered when α-Syn samples were incubated in the presence of AS69, providing a promissing agent for early interference with the pathogenesis of synucleinopathies [116].

Following these emerging trends in protein engineering, the SLS-007 peptide family targeting the α-Syn NAC domain (residues 68–78) was designed [233,234]. The proposed mechanism of their activity is related to the seeding pathways of α-Syn and other aggregation-prone proteins. The structures of two SLS-007 peptide family peptides, S62 and S71, are complementary to the α-Syn fibril core. In combination with the adeno-associated virus as a vector delivery system, these peptides were subjected to a preclinical trial [234].

To prevent α-Syn aggregation and elongation of its fibrils, Ventura’s team [101] recently searched for a peptide complementary to the highly exposed hydrophobic α-Syn core and negative charges concentrated at the C-terminal tails of “fuzzy coat” present in two types of α-Syn oligomers. Using a structure-guided approach and high throughput screening, they identified a human peptide, PSMα3, expressed in the brain and the gastrointestinal tract containing 22 amino acids and bearing a short, stable, amphipathic, and cationic helical conformation [101,130]. PSMα3 exhibited negligible anti-aggregative activity towards α-Syn. However, it bound α-Syn toxic oligomers and fibrils with nanomolar affinity, causing substoichiometric inhibition of α-Syn aggregation and cancelling the oligomer-induced damage in neuronal cell models. PSMα3 showed stronger α-Syn amyloid aggregation inhibitory potential than the above-mentioned SynuClean-D, previously discovered by the same team [190].

In the panel of disease-modifying therapies, the application of small peptides to control α-Syn oligomerization has been on the rise [235]. Moreover, the interest in peptides as therapeutic agents in neurodegenerative diseases linked with amyloidopathies is increasing due to improvements in delivery strategies, manufacture of large peptide libraries, synthetic viability, and high-throughput screening [222]. Taken together, protein engineering and selecting endogenous peptides showing analogous binding, anti-aggregation, and detoxifying properties towards α-Syn monomers and oligomers opens previously unexplored space for PD diagnosis and/or therapies.

#### 4.2.4. Clearance and Degradation of Toxic α-Syn Aggregates

Besides biosynthesis, folding, and PTMs, α-Syn amount depends on its degradation and clearance via autophagy (dominant-type lysosome) and the proteasome/ubiquitin system (Figure 3). The α-Syn monomer is normally degraded by lysosomes through chaperone-mediated autophagy, while wildtype and mutated α-Syn aggregates exhibiting a longer half-life are degraded by macroautophagy. Among mutated genes related to PD development, nineteen are associated with mitophagy, macroautophagy, chaperone-mediated autophagy, and lysosomes [158]. Particularly, mutations in genes encoding lysosomal hydrolases and their delivery (e.g., *GBA1* encoding acid-β-glucosidase, glucocerebrosidase), lysosome acidification (e.g., *ATP6AP2* encoding ATPase H^+^ transporting accessory protein 2 and *ATP13A2* encoding ATPase 13A2 cation transporting), and lysosomal ion channels or transporters (*TMEM175* encoding lysosomal K^+^ channel) are associated with PD development [236,237,238]. Additionally, disturbance within the multifaceted cellular clearance mechanisms and correlated transport processes leads to the spreading of the toxic α-Syn forms through the synaptic space to adjacent neurons and glial cells [239]. Furthermore, misfolded α-Syn causes clearance impairment through the overload of chaperone-mediated autophagy and reduced lysosomal enzyme activity [240,241]. Autophagy impairment has also been observed in various neurodegenerative conditions with similar pathophysiology of proteinaceous inclusion formation.

One of the emerging PD therapy approaches is based on boosting toxic α-Syn species clearance and degradation [25,58]. Reducing aggregation can be accomplished by hindering α-Syn multimerisation through molecular chaperones. These proteins assist with proper protein folding and the clearance of misfolded proteins and toxic aggregates. Two molecular chaperones have been used to block amyloid-β fibril ends and the fibril surfaces of amyloid-β protein in Alzheimer’s disease [242]. Results showed that both molecular chaperones exhibited nucleation inhibition, but at different steps of the process, indicating the fine-tuning of these reparation pathways. 

Interestingly, several heat shock proteins (HSPs, members of the chaperone protein family group) such as HSP104, HSP70, and HSP40 co-localise with α-Syn in LBs. HSP104 can inhibit α-Syn aggregation and disassemble α-Syn oligomers and fibrils in vitro [52]. HSP70 together with HSP40 participate in the ubiquitination and proteasomal degradation of α-Syn [243]. Overexpression of HSP70 showed beneficial effects in Drosophila and transgenic mouse PD model systems [52]. HSP70 inhibited α-Syn aggregation by binding to the oligomer’s hydrophobic core and stabilizing its non-toxic-disordered conformation [244]. On the other side, HSP90 stabilises proteins and prevents their ubiquitination. Pertinent to this, one successful therapeutic approach was based on tight α-Syn proteostasis regulation by HSP90 activity inhibition or/and by HSP70 activity stimulation resulting in increased α-Syn degradation [245,246]. Despite these encouraging results, we need to better understand their interaction with other proteins involved in PD pathology (especially with PINK1, Parkin, and DJ-1) that are related to oxidative stress and proteostasis [247]. The additional challenges of this approach are related to HSP overexpression, specific HSP selection, and their interaction (especially regarding HSP27, HSP 70, and HSP 104), as well as their degradation capacity and limitations regarding the level of abnormal α-Syn accumulation [248,249,250]. 

Regarding, the autophagy–lysosomal system, aspartic-protease cathepsin D is the major protease involved in α-Syn degradation. The α-Syn aggregates were observed in cathepsin D-deficient mice, whereas in transgenic mice overexpressing cathepsin D, α-Syn aggregate accumulation was diminished, protecting dopaminergic neuronal cells from damage [251]. Besides hydrolases, pH is the most critical variable that can dramatically influence the activity of soluble lysosomal hydrolases (including cathepsin D) as well as lysosomal membrane proteins that are critical for lysosome activity [252]. A multimeric, ATP-driven proton pump, v-ATPase, is responsible for controlling the endosomal and lysosomal pH [236]. In addition to H^+^, some lysosomal enzymes require a specific amount of Ca^2+^, Fe^2+^, and Zn^2+^, regulated by various cation channels, including transient receptor potential mucolipin channels 1/3 (TRPML1/3), and transporters, such as Zn^2+^ transporters 2 and 4, and ATP13A2, also known as PARK9 [237]. High lysosomal pH leads to aberrant Ca^2+^ efflux from lysosomal TRPML1 channels, causing a lysosomal acidification deficit [236]. Thereby, it is not surprising that various specific agonists or antagonists of autophagic activity targeting these factors have already been identified (e.g., by high-throughput screening) as candidates for development and clinical studies, and some have even reached the market. These candidates include lysosomal acidification inhibitors (chloroquine, hydroxychloroquine), cathepsin D inhibitors (pepstatin A), v-ATPase inhibitors (bafilomycin A1, saliphenylhalamide), TRPML1/3 agonists, and lysosomal activators (lonafarnib) [237]. In addition, previously described α-Syn natural disaggregators such as curcumin, baicalein, kaempferol, spermidine, trehalose, terpenoids paeoniflorin, celastrol, and onjisaponin B are also among the candidates that might stimulate clearance and degradation of toxic α-Syn forms [158]. 

Opposite to previously described studies related to intracellular clearance of different α-Syn species, several studies have focused on mechanisms of extracellular α-Syn oligomer degradation (reviewed in Stefanis et al. [253]). As mentioned, extracellular α-Syn aggregates can exhibit neurotoxic and neuroinflammatory activity as well as neuron-to-neuron spreading pathology [138]. Degradation of extracellular α-Syn forms can be based on cell-mediated degradation involving endocytosis and autophagosome-related degradation, and on extracellular enzyme-based hydrolysis. Among identified proteases involved in extracellular α-Syn hydrolysis, serine proteases neurosin and plasmin, and matrix metalloproteinases (induced by oxidative damage) are studied the most [253]. To be considered in drug development for PD, the role of these proteases in various cellular pathways and their interactome needs to be better understood.

#### 4.2.5. Capturing Toxic α-Syn Aggregates and Blocking Transcellular Spreading

Toxic forms of α-Syn can be captured by therapeutic antibodies against α-Syn N- and C-terminal regions in the presynaptic space or extracellularly (Figure 3) [59]. Given the neuron-to-neuron transfer of the α-Syn toxic forms and their transient presence in extracellular space, the odds of binding to a specific antibody increase.

Monoclonal antibodies are mainly directed against the C-terminus, but some target the epitope near the N-terminus of the α-Syn monomer, as well as the protein oligomers and protofibrils [25]. The most extensively studied is prasinezumab humanized IgG1 monoclonal antibody directed against epitopes near the C-terminus (Hoffmann–La Roche–Prothen). Prasinezumab showed dose-dependent lowering of the monomeric α-Syn amount in the serum and good safety and tolerance [254,255]. In preclinical studies, prasinezumab reduced neurodegeneration, as evidenced by decreased α-Syn aggregation and spreading in the α-Syn-transgenic mouse PD model [256]. Currently, the second phase of the clinical trials is ongoing, aiming to investigate the efficacy of this antibody in 300 patients with early-stage PD who have not received levodopa (NCT03100149, NCT04777331) [25]. Furthermore, human-derived α-Syn antibody BIIB054 (Biogen) prevented toxic α-Syn aggregate transmission in mice [257] and is further directed to a clinical trial phase. At the moment, there are four more candidates for passive immunization in different stages of clinical and preclinical trials [25]. These early trials have demonstrated good tolerability, though antibody distribution in plasma was higher than in CSF, lowering their efficiency [33].

The main challenges in neuro-immunotherapies are related to smidgen amounts of intracellular α-Syn, as well as poor BBB penetrability, mostly due to a high molecular weight of antibodies. To overcome this obstacle, an interesting emerging research avenue relies on gene-engineered antibodies, called intrabodies [88]. Intrabodies are small, 14–30 kDa proteins that are generated from antibody fragments of Fv variable regions, designed to act at the intracellular level [168]. There are several intrabody types depending on combinations of heavy and light immunoglobulin chains. Structures based on small, heavy-chain fragments of immunoglobulins are called nanobodies [258]. Two recent studies reported promising results regarding intrabodies targeting the α-Syn NAC and C-terminal regions [259,260]. Their beneficial effect is based on impeding α-Syn aggregation and the ability to decrease proteasomal stress, decreasing α-Syn accumulation [168]. The provided data indicate promising potential for immunization to support PD treatment, particularly considering the progress in bioinformatic and gene therapy, as well as improvements in diagnostics [258].

Considering the shortcomings of passive immunization, there is a need to develop active immunotherapy and to involve humoral immune response. At the moment, three vaccines, PD01A, PD03A, and UB-132 (Affiris), are being evaluated clinically due to their neuroprotective impact and positive safety response [139,261,262]. PD01A is directed at the α-Syn C-terminus, while PD03A targets 12 amino acid residues near the C-terminus [261,263]. PD01A exhibited a positive effect on the reduction of α-Syn aggregation, as well as on cognitive and motor symptoms, satisfying safety and tolerance requirements. PD03A showed a response similar to that of PD01A. UB-132-produced antibodies bind α-Syn oligomers and fibrils in guinea pigs, while research related to its neurophysiological role is ongoing [25,261]. Innovation in vaccine design requires simultaneous nanotechnology application to develop novel delivery systems. These approaches involve merging both immune system aspects in order to achieve the optimal response and neuroprotection [264]. Although both neuro-immunotherapy types present elegant tools to inhibit the pathogenic spread of extracellular aggregated α-Syn, the associated risks, such as systemic side reactions, need to be fully explored [88].

The use of receptor-neutralising strategies to inhibit α-Syn internalization and transcellular propagation would be an interesting therapeutic avenue. As mentioned, toxic α-Syn forms can escape the original neuron and transfer to the synaptic space, followed by receptor-mediated endocytosis of the adjacent cell, resulting in the advancement of Lewy pathology. Since interaction between the α-Syn C-terminus and LAG3 receptor facilitate its cellular uptake, by blocking this interaction, α-Syn transmission would cease. Expectedly, the application of LAG3-directed antibodies significantly reduces misfolded α-Syn-induced toxicity and spreading [265]. In addition, TLR2-neutralizing by the anti-TLR2 antibody blocks neuron-to-neuron and neuron-to-astrocyte α-Syn transmission in vitro and alleviates neuroinflammation, neurodegeneration, and behavioural deficits in an α-Syn transgenic mouse PD model [266]. On the other hand, disruptors of heparan sulphate proteoglycans reduced endocytic α-Syn uptake [136]. Further experiments should be conducted in animal models to identify specific inhibitors of heparan sulphate proteoglycans that can slow the pathology propagation cycle without interfering with essential cellular processes.

## 5. Conclusions and Further Perspectives

PD affects ~1–2% of people over 65 years of age, with a slight effect based on sex/gender and socioeconomic status. Despite numerous studies, the cure for PD has not yet been found. Current therapies alleviate early motor symptoms, but they are not helpful as the pathology progresses. This highlights the urgency of discovering new approaches for earlier diagnosis, treatment, and especially prevention or slowing of neurodegeneration associated with PD and related synucleinopathies. 

The multifactorial nature of PD and other synucleinopathies and the partial understanding of key molecular events and neurotoxic species that accumulate during α-Syn misfolding and aggregation are among the major obstacles to finding a cure. Currently, the molecular mechanisms and factors modulating α-Syn aggregation remain obscure, highlighting the need for further studies. According to the National Institute of Neurological Disorders and Stroke [267], a better understanding of the normal and abnormal functions of α-Syn is a key step in the promotion of novel disease-modulating therapeutic strategies.

Although much has been revealed regarding structure, function, misfunction, and aggregation, the complete puzzle is still not solved. Innovative studies targeting α-Syn and its oligomers and fibrils are gaining momentum. The intrinsically disordered nature of α-Syn and its dynamic behaviour and conformational plasticity, together with the wide spectrum of interactions, present the main challenge in developing α-Syn-centric approaches, particularly those based on molecular recognition and identifying compounds with α-Syn anti-aggregation potential. Thus, in our opinion, the most promising PD therapeutic strategies rely on the rational design of peptides and/or peptidomimetics. Despite the benefits associated with peptides, namely high affinity and selectivity among biomolecules, their poor serum stability and low BBB permeability threatens their application. These hindrances can be overcome by chemical modifications, such as glycoside addition and the replacement of relevant amino acids with D-analogues. 

Regarding the specific candidates that can directly alter the α-Syn aggregation pathway, it is important to answer the following questions:

1. Which species in the α-Syn aggregation pathway (monomer, oligomers, preformed fibrils) is the best target?
2. Could selected disaggregators be used in combination with other ongoing approaches (gene silencing, immunotherapy, clearance stimulation) to achieve better efficiency in disease progress modification?

Selected candidates have to accumulate in the right concentration and location to efficiently prevent α-Syn misfolding. Obviously, the stability, susceptibility to proteolysis, immunogenicity, and cell toxicityof the proposed candidates have to be validated. To fulfil these requirements, we suggest potential peptides that can stabilise native α-Syn’s structure and membranes should be searched within naturally existing peptides/proteins that are known to protect cellular biomolecules under adverse conditions. Peptides/peptidomimetics that effectively prevent or significantly reduce α-Syn aggregation should represent proof-of-concept molecules for future studies aimed at improving their scaffolds to cross the BBB and for intracellular targeting. 

## Data Availability

Not applicable.

## References

[1] Giorgetti S., Greco C., Tortora P., Aprile F.A. (2018). Targeting amyloid aggregation: An overview of strategies and mechanisms. Int. J. Mol. Sci..

[2] Gao J., Perera G., Bhadbhade M., Halliday G.M., Dzamko N. (2019). Autophagy activation promotes clearance of alpha-synuclein inclusions in fibril-seeded human neural cells. J. Biol. Chem..

[3] Wang Z., Gao G., Duan C., Yang H. (2019). Progress of immunotherapy of anti-α-synuclein in Parkinson’s disease. Biomed. Pharm..

[4] Zharikov A., Bai Q., De Miranda B.R., Van Laar A., Greenamyre J.T., Burton E.A. (2019). Long-term RNAi knockdown of α-synuclein in the adult rat *substantia nigra* without neurodegeneration. Neurobiol. Dis..

[5] Kouli A., Torsney K.M., Kuan W.L., Stoker T.B., Greenland J.C. (2018). Parkinson’s disease: Etiology, neuropathology, and pathogenesis. Parkinson’s Disease: Pathogenesis and Clinical Aspects.

[6] Meade R.M., Fairlie D.P., Mason J.M. (2019). Alpha-synuclein structure and Parkinson’s disease—Lessons and emerging principles. Mol. Neurodegener..

[7] Dorsey E.R., Elbaz A., Nicjols E., Abbasi N., Abd-Allah F., Abdelalim A., Adsur J.C., Ansha M.G., Brayne C., Choi J.Y.J. (2018). Global, regional, and national burden of Parkinson’s disease, 1990–2016: A systematic analysis for the global burden of disease study. Lancet Neurol..

[8] Ball N., Teo W.P., Chandra S., Chapman J. (2019). Parkinson’s disease and the environment. Front. Neurol..

[9] European Parkinson Disease Association (2011). The European PD Standards of Care Consensus Statement. https://www.epda.eu.com/latest/resources/the-european-parkinsons-disease-standards-of-care-consensus-statement/.

[10] Ou Z., Pan J., Tang S., Duan D., Yu D., Nong H., Wang Z. (2021). Global trends in the incidence, prevalence, and years lived with disability of Parkinson’s disease in 204 countries/territories from 1990 to 2019. Front. Public Health.

[11] Abbott R.D., Petrovitch H., White L.R., Masaki K.H., Tanner C.M., Curb J.D., Grandinetti A., Blanchette P.L., Popper J.S., Ross G.W. (2001). Frequency of bowel movements and the future risk of Parkinson’s disease. Neurology.

[12] Jost W.H. (2010). Gastrointestinal dysfunction in Parkinson’s disease. J. Neurol. Sci..

[13] Schaeffer E., Kluge A., Böttner M., Zunke F., Cossais F., Berg D., Arnold P. (2020). Alpha synuclein connects the gut-brain axis in Parkinson’s disease patients—A view on clinical aspects. Cellular pathology and analytical methodology. Front. Cell Dev. Biol..

[14] Kalia L.V., Lang A.E. (2015). Parkinson’s disease. Lancet.

[15] Armstrong M.J., Okun M.S. (2020). Diagnosis and treatment of Parkinson’s disease: A review. JAMA.

[16] He S., Zhong S., Liu G., Yang J. (2020). Alpha-synuclein: The interplay of pathology, neuroinflammation, and environmental factors in Parkinson’s disease. Neurodegener. Dis..

[17] Ganguly U., Singh S., Pal S., Prasad S., Agrawal B.K., Saini R.V., Chakrabarti S. (2021). Alpha-synuclein as a biomarker of Parkinson’s disease: Good, but not good enough. Front. Aging Neurosci..

[18] Yang W., Hamilton J.L., Kopil C., Beck J.C., Tanner C.M., Albin R.L., Dorsey R., Dahodwala N., Cintina I., Hogan P. (2020). Current and projected future economic burden of Parkinson’s disease in the US. NPJ Parkinsons Dis..

[19] World Health Organisation, World Federation of Neurology (2017). Atlas. Country Resources for Neurological Disorders.

[20] Muddapu V.R., Chakravarthy V.S. (2021). Influence of energy deficiency on the subcellular processes of Substantia Nigra Pars Compacta cell for understanding Parkinsonian neurodegeneration. Sci. Rep..

[21] Spillantini M.G., Schmidt M.L., Lee V.M., Trojanowski J.Q., Jakes R., Goedert M. (1997). Alpha-synuclein in Lewy bodies. Nature.

[22] Parmar M., Grealish S., Henchcliffe C. (2020). The future of stem cell therapies for Parkinson’s disease. Nat. Rev. Neurosci..

[23] Twohig D., Nielsen H.M. (2019). α-synuclein in the pathophysiology of Alzheimer’s disease. Mol. Neurodegener..

[24] Brás I.C., Outeiro T.F. (2021). Alpha-synuclein: Mechanisms of release and pathology progression in synucleinopathies. Cells.

[25] Grosso Jasutkar H., Oh S.E., Mouradian M.M. (2022). Therapeutics in the pipeline targeting α-synuclein for Parkinson’s disease. Pharmacol. Rev..

[26] Polymeropoulos M.H., Lavedan C., Leroy E., Ide S.E., Dehejia A., Dutra A., Pike B., Root H., Rubenstein J., Boyer R. (1997). Mutation in the alpha-synuclein gene identified in families with Parkinson’s disease. Science.

[27] Maraganore D.M., De Andrade M., Elbaz A., Farrer M.J., Ioannidis J.P., Krüger R., Walter A.R., Schneider N.K., Lesnick T.G., Lincoln S.J. (2006). Genetic Epidemiology of Parkinson’s Disease (GEO-PD) Consortium. Collaborative analysis of α-synuclein gene promoter variability and Parkinson’s disease. JAMA.

[28] Soldner F., Stelzer Y., Shivalila C.S., Abraham B.J., Latourelle J.C., Barrasa M.I., Goldmann J., Myers R.H., Young R.A., Jaenisch R. (2016). Parkinson-associated risk variant in distal enhancer of α-synuclein modulates target gene expression. Nature.

[29] Singleton A.B., Farrer M., Johnson J., Singleton A., Hague S., Kachergus J., Hulihan M., Peuralinna T., Dutra A., Nussbaum R. (2003). Alpha-synuclein locus triplication causes Parkinson’s disease. Science.

[30] Chartier-Harlin M.C., Kachergus J., Roumier C., Mouroux V., Douay X., Lincoln S., Levecque C., Larvor L., Andrieux J., Hulihan M. (2004). Alpha-synuclein locus duplication as a cause of familial Parkinson’s disease. Lancet.

[31] Wong Y.C., Krainc D. (2017). Alpha-synuclein toxicity in neurodegeneration: Mechanism and therapeutic strategies. Nat. Med..

[32] Trinh J., Zeldenrust F.M.J., Huan J., Kasten M., Schaake S., Petkovic S., Madoev H., Grünewald A., Almuammar S., König I.R. (2018). Genotype-phenotype relations for the Parkinson’s disease genes SNCA, LRRK2, VPS35: MDS Gene systematic review. Mov. Disord..

[33] Sardi S.P., Viel C., Clarke J., Treleaven C.M., Richards A.M., Park H., Olszewski M.A., Dodge J.C., Marshall J., Makino E. (2017). Glucosylceramide synthase inhibition alleviates aberrations in synucleinopathy models. Proc. Natl. Acad. Sci. USA.

[34] Grova N., Schroeder H., Olivier J.-L., Turner J.D. (2019). Epigenetic and neurological impairments associated with early life exposure to persistent organic pollutants. Int. J. Genom..

[35] Jowaed A., Schmitt I., Kaut O., Wüllner U. (2010). Methylation regulates alpha-synuclein expression and is decreased in Parkinson’s disease patients’ brains. J. Neurosci..

[36] Ascherio A., Schwarzschild M.A. (2016). The epidemiology of Parkinson’s disease: Risk factors and prevention. Lancet Neurol..

[37] Wassouf Z., Schulze-Hentrich J.M. (2019). Alpha-synuclein at the nexus of genes and environment: The impact of environmental enrichment and stress on brain health and disease. J. Neurochem..

[38] Chin-Chan M., Navarro-Yepes J., Quintanilla-Vega B. (2015). Environmental pollutants as risk factors for neurodegenerative disorders: Alzheimer’s and Parkinson’s diseases. Front. Cell Neurosci..

[39] Narayan S., Liew Z., Bronsteinb J.M., Ritz B. (2017). Occupational pesticide use and Parkinson’s disease in the Parkinson environment gene (PEG). Study Environ. Int..

[40] Ferreira C., Almeida C., Tenreiro S., Quintas A. (2020). Neuroprotection or neurotoxicity of illicit drugs on Parkinson’s disease. Life.

[41] Ahmed H., Abushouk A.I., Gabr M., Negida A., Abdel-Daim M.M. (2017). Parkinson’s disease and pesticides: A meta analysis of disease connection and genetic alterations. Biomed. Pharmacother..

[42] Islam M.D., Azim F., Saju H., Zargaran A., Shirzad M., Kamal M., Fatema K., Rehman S., Momith Azad M.A., Ebrahimi-Barough S. (2021). Pesticides and Parkinson’s disease: Current and future perspective. J. Chem. Neuroanat..

[43] Manning-Bog A.B., McCormack A.L., Li J., Uversky V.N., Fink A.L., Di Monte D.A. (2002). The herbicide paraquat causes up-regulation and aggregation of alpha-synuclein in mice: Paraquat and alpha-synuclein. J. Biol. Chem..

[44] Sherer T.B., Kim J.H., Betarbet R., Greenamyre J.T. (2003). Subcutaneous rotenone exposure causes highly selective dopaminergic degeneration and alpha-synuclein aggregation. Exp. Neurol..

[45] Uversky V.N., Li J., Fink A.L. (2001). Pesticides directly accelerate the rate of alpha-synuclein fibril formation: A possible factor in Parkinson’s disease. FEBS Lett..

[46] Kaidery N.A., Tarannum S., Thomas B. (2013). Epigenetic landscape of Parkinson’s disease: Emerging role in disease mechanisms and therapeutic modalities. Neurotherapeutics.

[47] Ritz B., Lee P.C., Hansen J., FuchLassen C., Ketzel M., Sørensen M., Raaschou-Nielsen O. (2016). Traffic-related air pollution and Parkinson’s disease in Denmark: A case-control study. Environ. Health Perspect..

[48] Calderón-Garcidueñas L., Solt A.C., Henríquez-Roldán C., Torres-Jardón R., Nuse B., Herritt L., Villarreal-Calderón R., Osnaya N., Stone I., García R. (2008). Long-term air pollution exposure is associated with neuroinflammation, an altered innate immune response, disruption of the blood-brain barrier, ultrafine particulate deposition, and accumulation of amyloid β-42 and α-synuclein in children and young adults. Toxicol. Pathol..

[49] Calderón-Garcidueñas L. (2021). Parkinson’s disease and air pollution: Does what we breathe matter?. Nat. Rev. Neurol..

[50] Kasdagli M.-I., Katsouyanni K., Dimakopoulou K., Samoli E. (2019). Air pollution and Parkinson’s disease: A systematic review and meta-analysis up to 2018. Int. J. Hyg. Environ. Health..

[51] Bjorklund G., Stejskal V., Urbina M.A., Dadar M., Chirumbolo S., Mutter J. (2018). Metals and Parkinson’s disease: Mechanisms and biochemical processes. Curr. Med. Chem..

[52] Breydo L., Wu J.W., Uversky V.N. (2012). α-Synuclein misfolding and Parkinson’s disease. Biochim. Biophys. Acta Mol. Basis Dis..

[53] Santner A., Uversky V.N. (2010). Metalloproteomics and metal toxicology of alpha-synuclein. Metallomics.

[54] Bisaglia M., Bubacco L. (2020). Copper ions and Parkinson’s disease: Why is homeostasis so relevant?. Biomolecules.

[55] Paik S.R., Shin H.J., Lee J.H., Chang C.S., Kim J. (1999). Copper (II)-induced self-oligomerization of alpha-synuclein. Biochem. J..

[56] Wei X., Cai M., Land J.L. (2021). The function of the metals in regulating epigenetics during Parkinson’s disease. Front. Genet..

[57] Weisskopf M.G., Weuve J., Nie H., Saint-Hilaire M.-H., Sudarsky L., Simon D.K., Hersh B., Schwartz J., Wright R.O., Hu H. (2010). Association of cumulative lead exposure with Parkinson’s disease. Environ. Health Perspect..

[58] Jankovic J., Tan E.K. (2020). Parkinson’s disease: Etiopathogenesis and treatment. J. Neurol. Neurosurg. Psychiatry.

[59] Kingwell K. (2017). Zeroing in on neurodegenerative α-synuclein. Nat. Rev. Drug. Discov..

[60] Ulmer T.S., Bax A., Cole N.B., Nussbaum R.L. (2005). Structure and dynamics of micelle-bound human α-synuclein. J. Biol. Chem..

[61] Georgieva E.R., Ramlall T.F., Borbat P.P., Freed J.H., Eliezer D. (2008). Membrane-bound α-synuclein forms an extended helix: Long-distance pulsed ESR measurements using vesicles, bicelles, and rodlike micelles. J. Amer. Chem. Soc..

[62] Kim T.D., Paik S.R., Yang C.H. (2002). Structural and functional implications of C-terminal regions of alpha-synuclein. Biochemistry.

[63] Piovesan D., Walsh I., Minervini G., Tosatto S.C.E. (2017). FELLS: Fast estimator of latent local structure. Bioinformatics.

[64] Gautier R., Douguet D., Antonny B., Drin G. (2008). HELIQUEST: A web server to screen sequences with specific alpha-helical properties. Bioinformatics.

[65] Mirdita M., Schütze K., Moriwaki Y., Heo L., Ovchinnikov S., Steinegger M. (2021). ColabFold-Making protein folding accessible to all. bioRxiv.

[66] Habchi J., Tompa P., Longhi S., Uversky V.N. (2014). Introducing protein intrinsic disorder. Chem. Rev..

[67] Bondos S.E., Dunker A.K., Uversky V.N. (2022). Intrinsically disordered proteins play diverse roles in cell signaling. Cell Commun. Signal..

[68] Zhang S., Liu Y.Q., Jia C., Lim Y.J., Feng G., Xu E., Long H., Kimura Y., Tao Y., Zhao C. (2021). Mechanistic basis for receptor-mediated pathological α-synuclein fibril cell-to-cell transmission in Parkinson’s disease. Proc. Natl. Acad. Sci. USA.

[69] Bertoncini C.W., Jung Y.S., Fernandez C.O., Hoyer W., Griesinger C., Jovin T.M., Zweckstetter M. (2005). Release of long-range tertiary interactions potentiates aggregation of natively unstructured alpha-synuclein. Proc. Natl. Acad. Sci. USA.

[70] Nuber S., Rajsombath M., Minakaki G., Winkler J., Müller C.P., Ericsson M., Caldarone B., Dettmer U., Selkoe D.J. (2018). Abrogating native α-synuclein tetramers in mice causes a L-DOPA-responsive motor syndrome closely resembling Parkinson’s disease. Neuron.

[71] Bartels T., Choi J.G., Selkoe D.J. (2011). Alpha-Synuclein occurs physiologically as a helically folded tetramer that resists aggregation. Nature.

[72] Wang W., Perovic I., Chittuluru J., Kaganovich A., Nguyen L.T., Liao J., Auclair J.R., Johnson D., Landeru A., Simorellis A.K. (2011). A soluble alpha-synuclein construct forms a dynamic tetramer. Proc. Natl. Acad. Sci. USA.

[73] Wang L., Das U., Scott D.A., Tang Y., McLean P.J., Roy S. (2014). α-Synuclein multimers cluster synaptic vesicles and attenuate recycling. Curr. Biol..

[74] Bendor J.T., Logan T.P., Edwards R.H. (2013). The function of alpha-synuclein. Neuron.

[75] Goers J., Manning-Bog A.B., McCormack A.L., Millett I.S., Doniach S., Di Monte D.A., Uversky V.N., Fink A.L. (2003). Nuclear localization of alpha-synuclein and its interaction with histones. Biochemistry.

[76] Takahashi M., Kanuka H., Fujiwara H., Koyama A., Hasegawa M., Miura M., Iwatsubo T. (2003). Phosphorylation of α-synuclein characteristic of synucleinopathy lesions is recapitulated in α-synuclein transgenic *Drosophila*. Neurosci. Lett..

[77] Siddiqui A., Chinta S.J., Mallajosyula J.K., Rajagopolan S., Hanson I., Rane A., Melov S., Andersen J.K. (2012). Selective binding of nuclear alpha-synuclein to the PGC1alpha promoter under conditions of oxidative stress may contribute to losses in mitochondrial function: Implications for Parkinson’s disease. Free Radic. Biol. Med..

[78] Ma K.L., Song L.K., Yuan Y.H., Zhang Y., Yang J.L., Zhu P., Chen N.H. (2014). Alpha-Synuclein is prone to interaction with the GC-box-like sequence in vitro. Cell Mol. Neurobiol..

[79] Pinho R., Paiva I., Jercic K.G., Fonseca-Ornelas L., Gerhardt E., Fahlbusch C., Garcia-Esparcia P., Kerimoglu C., Pavlou M.A.S., Villar-Piqué A. (2019). Nuclear localization and phosphorylation modulate pathological effects of alpha-synuclein. Hum. Mol. Genet..

[80] Bernal-Conde L.D., Ramos-Acevedo R., Reyes-Hernández M.A., Balbuena-Olvera A.J., Morales-Moreno I.D., Argüero-Sánchez R., Schüle B., Guerra-Crespo M. (2020). Alpha-synuclein physiology and pathology: A perspective on cellular structures and organelles. Front. Neurosci..

[81] Burré J., Sharma M., Sudhof T.C. (2018). Cell biology and pathophysiology of α-synuclein. Cold Spring Harb. Perspect. Med..

[82] Burré J., Sharma M., Tsetsenis T., Buchman V., Etherton M.R., Sudhof T.C. (2010). Alpha-synuclein promotes SNARE-complex assembly in vivo and in vitro. Science.

[83] Campioni S., Riek R. (2016). Membrane remodelling activity of α-synuclein. J. Neurol. Neuromed..

[84] Sun J., Wang L., Bao H., Premi S., Das U., Chapman E.R., Roy S. (2019). Functional cooperation of alpha-synuclein and VAMP2 in synaptic vesicle recycling. Proc. Natl. Acad. Sci. USA.

[85] DeWitt D.C., Rhoades E. (2013). Alpha-synuclein can inhibit SNARE-mediated vesicle fusion through direct interactions with lipid bilayers. Biochemistry.

[86] Burré J., Sharma M., Südhof T.C. (2014). α-Synuclein assembles into higher-order multimers upon membrane binding to promote SNARE complex formation. Proc. Natl. Acad. Sci. USA.

[87] Payton J.E., Perrin R.J., Woods W.S., George J.M. (2004). Structural determinants of PLD2 inhibition by α-synuclein. J. Mol. Biol..

[88] Fields C.R., Bengoa-Vergniory N., Wade-Martins R. (2019). Targeting alpha-synuclein as a therapy for Parkinson’s disease. Front. Mol. Neurosci..

[89] Dalfo E., Ferrer I. (2005). α-synuclein binding to rab3a in multiple system atrophy. Neurosci. Lett..

[90] Paciotti S., Bellomo G., Gatticchi L., Parnetti L. (2018). Are we ready for detecting α-synuclein prone to aggregation in patients? The case of “protein-misfolding cyclic amplification” and “real-time quaking-induced conversion” as diagnostic tools. Front. Neurol..

[91] Del Tredici K., Braak H. (2016). Sporadic Parkinson’s disease: Development and distribution of alpha-synuclein pathology. Neuropathol. Appl. Neurobiol..

[92] Kuo Y.-M., Nwankwo E.I., Nussbaum R.L., Rogers J., Maccecchini M.L. (2019). Translational inhibition of α-synuclein by posiphen normalizes distal colon motility in transgenic Parkinson mice. Am. J. Neurodegener. Dis..

[93] Karpowicz R.J., Trojanowski J.Q., Lee V.M. (2019). Transmission of alpha-synuclein seeds in neurodegenerative disease: Recent developments. Lab. Investig..

[94] Böttner M., Fricke T., Müller M., Barrenschee M., Deuschl G., Schneider S.A., Egberts J.-H., Becker T., Fritscher-Ravens A., Ellrichmann M. (2015). Alpha-synuclein is associated with the synaptic vesicle apparatus in the human and rat enteric nervous system. Brain Res..

[95] Bu L.-L., Huang K.-X., Zheng D.-Z., Lin D.-Y., Chen Y., Jing X.-N., Liang Y.-R., Tao E.-X. (2020). Alpha-synuclein accumulation and its phosphorylation in the enteric nervous system of patients without neurodegeneration: An explorative study. Front. Aging Neurosci..

[96] Martins M., Rosa A., Guedes L.C., Fonseca B.V., Gotovac K., Violante S., Mestre T., Coelho M., Rosa M.M., Martin E.R. (2011). Convergence of miRNA expression profiling, alpha-synuclein interacton and GWAS in Parkinson’s disease. PLoS ONE.

[97] Alam P., Bousset L., Melki R., Otzen D.E. (2019). Alpha-synuclein oligomers and fibrils: A spectrum of species, a spectrum of toxicities. J. Neurochem..

[98] Burré J. (2015). The synaptic function of alpha-synuclein. J. Parkinson’s Dis..

[99] Shahmoradian S.H., Lewis A.J., Genoud C., Hench J., Moors T.E., Navarro P.P., Castaño-Díez D., Schweighauser G., Graff-Meyer A., Goldie K.N. (2019). Lewy pathology in Parkinson’s disease consists of crowded organelles and lipid membranes. Nat. Neurosci..

[100] Fusco G., Chen S.W., Williamson P.T., Cascella R., Perni M., Jarvis J.A., Cecchi C., Vendruscolo M., Chiti F., Cremades N. (2017). Structural basis of membrane disruption and cellular toxicity by α-synuclein oligomers. Science.

[101] Santos J., Gracia P., Navarro S., Peña-Díaz S., Pujols J., Cremades N., Pallarès I., Ventura S. (2021). α-Helical peptidic scaffolds to target α-synuclein toxic species with nanomolar affinity. Nat. Commun..

[102] Uversky V.N., Cooper E.M., Bower K.S., Li J., Fink A.L. (2002). Accelerated alpha-synuclein fibrillation in crowded milieu. FEBS Lett..

[103] Beyer K. (2006). Alpha-synuclein structure, posttranslational modification and alternative splicing as aggregation enhancers. Acta Neuropathol..

[104] Schmidt S., Vogt Waisenhorn D.M., Wurs W. (2022). Chapter 5—“Parkinson’s disease—A role of non-enzymatic posttranslational modifications in disease onset and progression?”. Mol. Aspect Med..

[105] Fujiwara H., Hasegawa M., Dohmae N., Kawashima A., Masliah E., Goldberg M.S., Shen J., Takio K., Iwatsubo T. (2002). Alpha-Synuclein is phosphorylated in synucleinopathy lesions. Nat. Cell Biol..

[106] Beyer K., Ariza A. (2013). α-Synuclein posttranslational modification and alternative splicing as a trigger for neurodegeneration. Mol. Neurobiol..

[107] Burai R., Ait-Bouziad N., Chiki A., Lashuel H.A. (2015). Elucidating the role of site-specific nitration of α-synuclein in the pathogenesis of Parkinson’s disease via protein semisynthesis and mutagenesis. J. Am. Chem. Soc..

[108] Zhang J., Li X., Jia-Da L. (2019). The roles of post-translational modifications on α-synuclein in the pathogenesis of Parkinson’s Diseases. Front. Neurosci..

[109] Burmann B.M., Gerez J.A., Matečko-Burmann I., Campioni S., Kumari P., Ghosh D., Mazur A., Aspholm E.E., Šulskis D., Wawrzyniuk M. (2020). Regulation of α-synuclein by chaperones in mammalian cells. Nature.

[110] Rott R., Szargel R., Shani V., Hamza H., Savyon M., Abd Elghani F., Bandopadhyay R., Engelender S. (2017). SUMOylation and ubiquitination reciprocally regulate α-synuclein degradation and pathological aggregation. Proc. Natl. Acad. Sci. USA.

[111] Krumova P., Meulmeester E., Garrido M., Tirard M., Hsiao H.H., Bossis G., Urlaub H., Zweckstetter M., Kügler S., Melchior F. (2011). Sumoylation inhibits alpha-synuclein aggregation and toxicity. J. Cell Biol..

[112] Abeywardana T., Pratt M.R. (2015). Extent of inhibition of α-synuclein aggregation in vitro by SUMOylation is conjugation site- and SUMO isoform-selective. Biochemistry.

[113] Marotta N.P., Lin Y.H., Lewis Y.E., Ambroso M.R., Zaro B.W., Roth M.T., Arnold D.B., Langen R., Pratt M.R. (2015). O-GlcNAc modification blocks the aggregation and toxicity of the protein α-synuclein associated with Parkinson’s disease. Nat. Chem..

[114] Celej M.S., Sarroukh R., Goormaghtigh E., Fidelio G.D., Ruysschaert J.M., Raussens V. (2012). Toxic prefibrillar *α*-synuclein amyloid oligomers adopt a distinctive antiparallel *β*-sheet structure. Biochem. J..

[115] Madine J., Doig A.J., Middleton D.A. (2008). Design of an N-methylated peptide inhibitor of alpha-synuclein aggregation guided by solid-state NMR. J. Am. Chem. Soc..

[116] Mirecka E.A., Shaykhalishahi H., Gauhar A., Akgul S., Lecher J., Willbold D., Stoldt M., Hoyer W. (2014). Sequestration of a beta-hairpin for control of alpha-synuclein aggregation. Angew. Chem..

[117] Zhao K., Li Y., Liu Z., Long H., Zhao C., Luo F., Sun Y., Tao Y., Su X.-D., Li D. (2020). Parkinson’s disease associated mutation E46K of α-synuclein triggers the formation of a distinct fibril structure. Nat. Commun..

[118] Guerrero-Ferreira R., Kovacik L., Ni D., Stahlberg H. (2020). New insights on the structure of alpha-synuclein fibrils using cryo-electron microscopy. Curr. Opin. Neurobiol..

[119] Tuttle M.D., Comellas G., Nieuwkoop A.J., Covell D.J., Berthold D.A., Kloepper K.D., Courtney J.M., Kim J.K., Barclay A.M., Kendall A. (2016). Solid-state NMR structure of a pathogenic fibril of full-length human alpha-synuclein. Nat. Struct. Mol. Biol..

[120] Yang X., Wang B., Hoop C.L., Williams J.K., Baum J. (2021). NMR unveils an N-terminal interaction interface on acetylated-α-synuclein monomers for recruitment to fibrils. Proc. Natl. Acad. Sci. USA.

[121] Zhao K., Lim Y.J., Liu Z., Long H., Sun Y., Hu J.J., Zhao C., Tao Y., Zhang X., Li D. (2020). Parkinson’s disease-related phosphorylation at Tyr39 rearranges α-synuclein amyloid fibril structure revealed by cryo-EM. Proc. Natl. Acad. Sci. USA.

[122] Guerrero-Ferreira R., Taylor N.M., Mona D., Ringler P., Lauer M.E., Riek R., Britschgi M., Stahlberg H. (2018). Cryo-EM structure of alpha-synuclein fibrils. eLife.

[123] Li B., Ge P., Murray K.A., Sheth P., Zhang M., Nair G., Sawaya M.R., Shin W.S., Boyer D.R., Ye S. (2018). Cryo-EM of full-length α-synuclein reveals fibril polymorphs with a common structural kernel. Nat. Commun..

[124] Li Y.W., Zhao C., Luo F., Liu Z., Gui X., Luo Z., Zhang X., Li D., Liu C., Li X. (2018). Amyloid fibril structure of α-synuclein determined by cryo-electron microscopy. Cell Res..

[125] Terada M., Suzuki G., Nonaka T., Kametani F., Tamaoka A., Hasegawa M. (2018). The effect of truncation on prion-like properties of α-synuclein. J. Biol. Chem..

[126] Bavinton C.E., Sternke-Hoffmann R., Knipe P.C., Yamashita T., Hamilton A.D., Luo J., Thompson S. (2022). Rationally designed helical peptidomimetics disrupt alpha-synuclein fibrillation. Chem. Commun..

[127] Conway K.A., Rochet J.C., Bieganski R.M., Lansbury P.T. (2001). Kinetic stabilization of the α-synuclein protofibril by a dopamine-α-synuclein adduct. Science.

[128] Padmaraju V., Bhaskar J.J., Prasada Rao U.J., Salimath P.V., Rao K.S. (2011). Role of advanced glycation on aggregation and DNA binding properties of alpha-synuclein. J. Alzheimer’s Dis..

[129] Ma K.L., Song L.K., Yuan Y.H., Zhang Y., Han N., Gao K., Chen N.H. (2014). The nuclear accumulation of alpha-synuclein is mediated by importin alpha and promotes neurotoxicity by accelerating the cell cycle. Neuropharmacology.

[130] Santos J., Pallarès I., Ventura S. (2022). Is a cure for Parkinson’s disease hiding inside us?. Trends Biochem. Sci.

[131] Angelova P.R., Ludtmann M.H., Horrocks M.H., Negoda A., Cremades N., Klenerman D., Dobson C.M., Wood N.W., Pavlov E.V., Gandhi S. (2016). Ca^2+^ is a key factor in α-synuclein-induced neurotoxicity. J. Cell Sci..

[132] Deas E., Cremades N., Angelova P.R., Ludtmann M.H., Yao Z., Chen S., Horrocks M.H., Banushi B., Little D., Devine M.J. (2016). Alpha-synuclein oligomers interact with metal ions to induce oxidative stress and neuronal death in Parkinson’s disease. Antioxid. Redox Signal..

[133] Ysselstein D., Joshi M., Mishra V., Griggs A.M., Asiago J.M., McCabe G.P., Stanciu L.A., Post C.B., Rochet J.C. (2015). Effects of impaired membrane interactions on α-synuclein aggregation and neurotoxicity. Neurobiol. Dis..

[134] Westphal C.H., Chandra S.S. (2013). Monomeric synucleins generate membrane curvature. J. Biol. Chem..

[135] Suzuki G., Imura S., Hosokawa M., Katsumata R., Nonaka T., Hisanaga S.I., Saeki Y., Hasegawa M. (2020). α-synuclein strains that cause distinct pathologies differentially inhibit proteasome. eLife.

[136] Holmes B.B., DeVos S.L., Kfoury N., Li M., Jacks R., Yanamandra K., Ouidja M.O., Brodsky F.M., Marasa J., Bagchi D.P. (2013). Heparan sulfate proteoglycans mediate internalization and propagation of specific proteopathic seeds. Proc. Natl. Acad. Sci. USA.

[137] Kim C., Ho D.-H., Suk J.-E., You S., Michael S., Kang J., Lee S.J., Masliah E., Hwang D., Lee H.-J. (2013). Neuron-released oligomeric alpha-synuclein is an endogenous agonist of TLR2 for paracrine activation of microglia. Nat. Commun..

[138] Danzer K.M., Haasen D., Karow A.R., Moussaud S., Habeck M., Giese A., Kretzschmar H., Hengerer B., Kostka M. (2007). Different species of alpha-synuclein oligomers induce calcium influx and seeding. J. Neurosci..

[139] Su R., Zhou T. (2021). Alpha-synuclein induced immune cells activation and associated therapy in Parkinson’s disease. Front. Aging Neurosci..

[140] Rostami J., Fotaki G., Sirois J., Mzezewa R., Bergström J., Essand M., Healy L., Erlandsson S. (2020). Astrocytes have the capacity to act as antigen-presenting cells in the Parkinson’s disease brain. J. Neuroinflam..

[141] Kwon S., Iba M., Masliah E., Kim C. (2019). Targeting microglial and neuronal Toll-like receptor 2 in synucleinopathies. Exp. Neurobiol..

[142] Kim C., Kwon S., Iba M., Spencer B., Rockenstein E., Mante M., Adame A., Shin S.J., Fields J.A., Robert A. (2021). Effects of innate immune receptor stimulation on extracellular α-synuclein uptake and degradation by brain resident cells. Exp. Mol. Med..

[143] Beach T.G., Serrano G.E., Kremer T., Canamero M., Dziadek S., Sade H., Derkinderen P., Corbillé A.G., Letournel F., Munoz D.G. (2018). Immunohistochemical method and histopathology judging for the systemic synuclein sampling study (S4). J. Neuropathol. Exp. Neurol..

[144] Stolzenberg E., Berry D., Yang D., Lee E.Y., Kroemer A., Kaufman S., Wong G.C.L., Oppenheim J.J., Sen S., Fishbein T. (2019). A role for neuronal alpha-synuclein in gastrointestinal immunity. J. Innate Immun..

[145] Tan E.-K., Chao Y.-X., West A., Chan L.-L., Poewe W., Jankovic J. (2020). Parkinson disease and the immune system—Associations, mechanisms and therapeutics. Nat. Rev. Neurol..

[146] Challis C., Hori A., Sampson T.R., Yoo B.B., Challis R.C., Hamilton A.M., Mazmanian S.K., Volpicelli-Daley L.A., Gradinaru V. (2020). Gut-seeded alpha-synuclein fibrils promote gut dysfunction and brain pathology specifically in aged mice. Nat. Neurosci..

[147] Kim S., Kwon S.H., Kam T.I., Panicker N., Karuppagounder S.S., Lee S., Lee J.H., Kim W.R., Kook M., Foss C.A. (2019). Transneuronal propagation of pathologic α-synuclein from the gut to the brain models Parkinson’s disease. Neuron.

[148] Breen D.P., Halliday G.M., Lang A.E. (2019). Gut-brain axis and the spread of α-synuclein pathology: Vagal highway or dead end?. Mov. Disord..

[149] Leclair-Visonneau L., Neunlist M., Derkinderen P., Lebouvier T. (2020). The gut in Parkinson’s disease: Bottom-up, top-down, or neither?. Neurogestroenerol. Motil..

[150] Du T., Wang L., Liu W., Zhu G., Chen Y., Zhang J. (2021). Biomarkers and the role of α-synuclein in Parkinson’s disease. Front. Aging Neurosci..

[151] Fayyad M., Salim S., Majbour N., Erskine D., Stoops E., Mollenhauer B., El-Agnaf O.M. (2019). Parkinson’s disease biomarkers based on α-synuclein. J. Neurochem..

[152] Alcalay R.N., Kehoe C., Shorr E., Battista R., Hall A., Simuni T., Marder K., Wills A.-M., Naito A., Beck J.C. (2020). Genetic testing for Parkinson disease: Current practice, knowledge, and attitudes among US and Canadian movement disorders specialists. Genet. Med..

[153] Musacchio J.M., Iverson L. (2013). Enzymes involved in the biosynthesis and degradation of catecholamines. Biochemistry of Biogenic Amines.

[154] Zahoor I., Shafi A., Haq E., Stoker T.B., Greenland J.C. (2018). Pharmacological treatment of Parkinson’s disease. Parkinson’s Disease: Pathogenesis and Clinical Aspects.

[155] Parkinson’s Disease Toolkit. http://www.toolkit.parkinson.org/content/first-line-meds-and-dosing.

[156] Nyholm D., Klangemo K., Johansson A. (2012). Levodopa/carbidopa intestinal gel infusion long-term therapy in advanced Parkinson’s disease. Eur. J. Neurol..

[157] Amjad F., Bhatti D., Davis T.L., Oguh O., Pahwa R., Kukreja P., Zamudio J., Metman L.V. (2019). Current practices for outpatient initiation of levodopa-carbidopa intestinal gel for management of advanced Parkinson’s disease in the United States. Adv. Ther..

[158] Bonam S.R., Tranchant C., Muller S. (2021). Autophagy-lysosomal pathway as potential therapeutic target in Parkinson’s disease. Cells.

[159] Athauda D., Gulyani S., Karnati H.K., Li Y., Tweedie D., Mustapic M., Chawla S., Chowdhury K., Skene S.S., Greig N.H. (2019). Utility of neuronal-derived exosomes to examine molecular mechanisms that affect motor function in patients with Parkinson disease. JAMA Neurol..

[160] Glotfelty E.J., Olson L., Karlsson T.E., Li Y., Greig N.H. (2020). Glucagon-like peptide-1 (GLP-1)-based receptor agonists as a treatment for Parkinson’s disease. Expert Opin. Invest Drugs.

[161] Barker R.A. (2019). Designing stem-cell-based dopamine cell replacement trials for Parkinson’s disease. Nat. Med..

[162] Christin C.W., Bankiewicz K.S., Van Laar A.D., Richardson R.M., Ravina B., Kells A.P., Boot B., Martin A.J., Nutt J., Thompson M.E. (2019). Magnetic resonance imaging guided phase 1 trial of putaminal AADC gene therapy for Parkinson’s disease. Ann. Neurol..

[163] Gonzalez R., Garitaonandia I., Semechkin A., Kern R. (2019). Derivation of neural stem cells from human parthenogenetic stem cells. Methods Mol. Biol..

[164] Jankovic J., Okun M.S., Kordower J.H. (2020). Stem cells: Scientific and ethical quandaries of a personalized approach to Parkinson’s disease. Mov. Disord..

[165] Teil M., Arotcarena M.-L., Faggiani E., Laferriere F., Bezard E., Dehay B. (2020). Targeting α-synuclein for PD therapeutics: A pursuit on all fronts. Biomolecules.

[166] Dansithong W., Paul S., Scoles D.R., Pulst S.M., Huynh D.P. (2015). Generation of SNCA cell models using zinc finger nuclease (ZFN) technology for efficient high throughput drug screening. PLoS ONE.

[167] Kantor B., Tagliafierro L., Gu J., Zamora M.E., Ilich E., Grenier C., Huang Z.Y., Murphy S., Chiba-Falek O. (2018). Downregulation of SNCA expression by targeted editing of DNA methylation: A potential strategy for precision therapy in PD. Mol. Ther..

[168] Mittal S., Bjørnevik K., Im D.S., Flierl A., Dong X., Locascio J.J., Abo K.M., Long E., Jin M., Xu B. (2017). B2-Adrenoreceptor is a regulator of the α-synuclein gene driving risk of Parkinson’s disease. Science.

[169] Gronich N., Abernethy D.R., Auriel E., Lavi I., Rennert G., Saliba W. (2018). β2- adrenoceptor agonists and antagonists and risk of Parkinson’s disease. Mov. Disord..

[170] Nielsen S.S., Gross A., Camacho-Soto A., Willis A.W., Racette B.A. (2018). B2-adrenoreceptor medications and risk of Parkinson’s disease. Ann. Neurol..

[171] Magistrelli L., Comi C. (2020). Beta2-adrenoceptor agonists in Parkinson’s disease and other synucleinopathies. J. Neuroimmune. Pharmacol..

[172] Hayashita-Kinoh H., Yamada M., Yokota T., Mizuno Y., Mochizuki H. (2006). Down-regulation of α-synuclein expression can rescue dopaminergic cells from cell death in the substantia nigra of Parkinson’s disease rat model. Biochem. Biophys. Res. Commun..

[173] Doxakis E. (2010). Post-transcriptional regulation of α-synuclein expression by mir-7 and mir-153. J. Biol. Chem..

[174] Zharikov A.D., Cannon J.R., Tapias V., Bai Q., Horowitz M.P., Shah V., El Ayadi A., Hastings T.G., Greenamyre J.T., Burton E.A. (2015). shRNA targeting α-synuclein prevents neurodegeneration in a Parkinson’s disease model. J. Clin. Investig..

[175] Takahashi M., Suzuki M., Fukuoka M., Fujikake N., Watanabe S., Murata M., Wada K., Nagai Y., Hohjoh H. (2015). Normalization of overexpressed α-synuclein causing Parkinson’s disease by a moderate gene silencing with RNA interference. Mol. Ther. Nucleic Acids..

[176] Alarócn-Arís D., Recasens A., Galofre M., Carballo-Carbajal I., Zacchi N., Ruiz-Bronchal E., Pavia-Collado R., Chica R., Ferres-Coy A., Santos M. (2018). Selective α-synuclein knockdown in monoamine neurons by intranasal oligonucleotide delivery: Potential therapy for Parkinson’s disease. Mol. Ther..

[177] Di Fusco D., Dinallo V., Marafini I., Figliuzzi M.M., Romano B., Monteleone G. (2019). Antisense oligonucleotide: Basic concepts and therapeutic application in inflammatory bowel disease. Front. Pharmacol..

[178] Dhuri K., Bechtold C., Quijano E., Pham H., Gupta A., Vikram A., Bahal R. (2020). Antisense oligonucleotides: An emerging area in drug discovery and development. J. Clin. Med..

[179] Cole T.A., Zhao H., Collier T.J., Sandoval I., Sortwell C.E., Steece-Collier K., Daley B.F., Booms A., Lipton J., Welch M. (2021). α-Synuclein antisense oligonucleotides as a disease-modifying therapy for Parkinson’s disease. JCI Insight.

[180] Gorbatyuk O.S., Li S., Nash K., Gorbatyuk M., Lewin A.S., Sullivan L.F., Mandel R.J., Chen W., Meyers C., Manfredsson F.P. (2010). In vivo RNAi-mediated alpha-synuclein silencing induces nigrostriatal degeneration. Mol. Ther..

[181] Khodr C.E., Becerra A., Han Y., Bohn M.C. (2014). Targeting alpha-synuclein with a microRNA-embedded silencing vector in the rat substantia nigra: Positive and negative effects. Brain Res..

[182] Benskey M.J., Sellnow R.C., Sandoval I.M., Sortwell C.E., Lipton J.W., Manfredsson F.P. (2018). Silencing alpha-synuclein in mature nigral neurons results in rapid neuroinflammation and subsequent toxicity. Front. Mol. Neurosci..

[183] Li H., Yang Y., Hong W., Huang M., Wu M., Zhao X. (2020). Applications of genome editing technology in the targeted therapy of human diseases: Mechanisms, advances and prospects. Signal. Transduct. Target. Ther..

[184] Reinhardt P., Schmid B., Burbulla L.F., Schöndorf D.C., Wagner L., Glatza M., Höing S., Hargus G., Heck S.A., Dhingra A. (2013). Genetic correction of a LRRK2 mutation in human iPSCs links parkinsonian neurodegeneration to ERK-dependent changes in gene expression. Cell Stem Cell.

[185] Disney M.D., Suresh B.M., Benhamou R.I., Childs-Disney J.L. (2020). Progress toward the development of the small molecule equivalent of small interfering RNA. Curr. Opin. Chem. Biol..

[186] Meyer S.M., Williams C.C., Akahori Y., Tanaka T., Aikawa H., Tong Y., Childs-Disney J.L., Disney M.D. (2020). Small molecule recognition of disease-relevant RNA structures. Chem. Soc. Rev..

[187] Zhou Z.D., Tan E.-K. (2017). Iron regulatory protein (IRP)-iron responsive element (IRE) signaling pathway in human neurodegenerative diseases. Mol. Neurodegener..

[188] Mikkilineni S., Cantuti-Castelvetri I., Cahill C.M., Balliedier A., Greig N.H., Rogers J.T. (2012). The anticholinesterase phenserine and its enantiomer posiphen as 5’untranslated-region-directed translation blockers of the Parkinson’s alpha synuclein expression. Parkinsons Dis..

[189] Lahiri D.K., Chen D., Maloney B., Holloway H.W., Yu Q.-S., Utsuki T., Giordano T., Sambamurti K., Greig N.H. (2007). The experimental Alzheimer’s disease drug posiphen [(1)-phenserine] lowers amyloid-β peptide levels in cell culture and mice. J. Pharmacol. Exp. Ther..

[190] Pujols J., Peña-Díaz S., Lázaro D.F., Peccati F., Pinheiro F., González D., Carija A., Navarro S., Conde-Giménez M., García J. (2018). Small molecule inhibits alpha-synuclein aggregation, disrupts amyloid fibrils, and prevents degeneration of dopaminergic neurons. Proc. Natl. Acad. Sci. USA.

[191] Sardi S.P., Cedarbaum J.M., Brundin P. (2018). Targeted therapies for Parkinson’s disease: From genetics to the clinic. Mov. Disord..

[192] Shihabuddin L.S., Brundin P., Greenamyre J.T., Stephenson D., Sardi S.P. (2018). New frontiers in Parkinson’s disease: From genetics to the clinic. J. Neurosci..

[193] Caruana M., Hogen T., Levin J., Hillmer A., Giese A., Vassallo N. (2011). Inhibition and disaggregation of alpha-synuclein oligomers by natural polyphenolic compounds. FEBS Lett..

[194] Toni M., Massimino M.L., De Mario A., Angiulli E., Spisni E. (2017). Metal dyshomeostasis and their pathological role in prion and prion-like diseases: The basis for a nutritional approach. Front. Neurosci..

[195] Ahmad B., Lapidus L.J. (2012). Curcumin prevents aggregation in α-synuclein by increasing reconfiguration rate. J. Biol. Chem..

[196] Ahsan N., Mishra S., Jain M.K., Surolia A., Gupta S. (2015). Curcumin pyrazole and its derivative (N-(3-nitrophenylpyrazole) curcumin inhibit aggregation, disrupt fibrils and modulate toxicity of wild type and mutant α-synuclein. Sci. Rep..

[197] Macedo D., Tavares L., McDougall G.J., Vicente Miranda H., Stewart D., Ferreira R.B., Santos C.N. (2015). (Poly)phenols protect from α-synuclein toxicity by reducing oxidative stress and promoting autophagy. Hum. Mol. Genet..

[198] Freyssin A., Page G., Fauconneau B., RiouxBilan A. (2018). Natural polyphenols effects on protein aggregates in Alzheimer’s and Parkinson’s prion-like diseases. Neural Regen. Res..

[199] Masuda M., Suzuki N., Taniguchi S., Oikawa T., Nonaka T., Iwatsubo T., Hasegawa M. (2006). Small molecule inhibitors of α-synuclein filament assembly. Biochemistry.

[200] Dominguez-Meijide A., Vasili E., Konig A., Cima-Omori M.S., Ibanez de Opakua A., Leonov A., Ryazanov S., Zweckstetter M., Griesinger C., Outeiro T.F. (2020). Effects of pharmacological modulators of alpha-synuclein and tau aggregation and internalization. Sci. Rep..

[201] Meng X., Munishkina L.A., Fink A.L., Uversky V.N. (2009). Molecular mechanisms underlying the flavonoid-induced inhibition of alpha-synuclein fibrillation. Biochemistry.

[202] Ono K., Tsuji M., Yamasaki T.R., Pasinetti G.M. (2020). Anti-aggregation effects of phenolic compounds on α-synuclein. Molecules.

[203] Kazakova O., Giniyatullina G., Babkov D., Wimmer Z. (2022). From marine metabolites to the drugs of the future: Squalamine, trodusquemine, their steroid and triterpene analogues. Int. J. Mol. Sci..

[204] Limbocker R., Staats R., Chia S., Ruggeri F.S., Mannini B., Xu C.K., Perni M., Cascella R., Bigi A., Sasser L.R. (2021). Squalamine and its derivatives modulate the aggregation of amyloid-β and α-synuclein and suppress the toxicity of their oligomers. Front. Neurosci..

[205] Perni M., Galvagnion C., Maltsev A., Meisl G., Muller M.B., Challa P.K., Kirkegaard J.B., Flagmeier P., Cohen S.I., Cascella R. (2017). A natural product inhibits the initiation of alpha-synuclein aggregation and suppresses its toxicity. Proc. Natl. Acad. Sci. USA.

[206] Perni M., Flagmeier P., Limbocker R., Cascella R., Aprile F.A., Galvagnion C., Heller G.T., Meisl G., Chen S.W., Kumita J.R. (2018). Multistep Inhibition of alpha-synuclein aggregation and toxicity in vitro and in vivo by trodusquemine. ACS Chem. Biol..

[207] Moree B., Yin G., Lázaro D.F., Munari F., Strohäker T., Giller K., Becker S., Outeiro T.F., Zweckstetter M., Salafsky J. (2015). Small molecules detected by second-harmonic generation modulate the conformation of monomeric α-synuclein and reduce its aggregation in cells. J. Biol. Chem..

[208] Fernandez C.O., Hoyer W., Zweckstetter M., Jares-Erijman E.A., Subramaniam V., Griesinger C., Jovin T.M. (2004). NMR of alpha-synuclein-polyamine complexes elucidates the mechanism and kinetics of induced aggregation. EMBO J..

[209] Wagner J., Ryazanov S., Leonov A., Levin J., Shi S., Schmidt F., Prix C., Pan-Montojo F., Bertsch U., Mitteregger-Kretzschmar G. (2013). Anle138b: A novel oligomer modulator for disease-modifying therapy of neurodegenerative diseases such as prion and Parkinson’s disease. Acta Neuropathol..

[210] Levin J., Schmidt F., Boehm C., Prix C., Botzel K., Ryazanov S., Leonov A., Griesinger C., Giese A. (2014). The oligomer modulator Anle138b inhibits disease progression in a Parkinson mouse model even with treatment started after disease onset. Acta Neuropathol..

[211] Pujols J., Peña-Díaz S., Conde-Giménez M., Pinheiro F., Navarro S., Sancho J., Ventura S. (2017). High-throughput screening methodology to identify alpha-synuclein aggregation inhibitors. Int. J. Mol. Sci..

[212] Peña-Díaz S., Pujols J., Vasili E., Pinheiro F., Santos J., Manglano-Artuñedo Z., Outeiro T.F., Ventura S. (2022). The small aromatic compound SynuClean-D inhibits the aggregation and seeded polymerization of multiple α-synuclein strains. J. Biol. Chem..

[213] Schrader T., Bitan G., Klärner F.G. (2016). Molecular tweezers for lysine and arginine–powerful inhibitors of pathologic protein aggregation. Chem. Commun..

[214] Attar A., Chan W.T., Klarner F.G., Schrader T., Bitan G. (2014). Safety and pharmacological characterization of the molecular tweezer CLR01—A broad-spectrum inhibitor of amyloid proteins’ toxicity. BMC Pharm. Toxicol..

[215] Hadrovic I., Rebmann P., Klarner F.G., Bitan G., Schrader T. (2019). Molecular lysine tweezers counteract aberrant protein aggregation. Front. Chem..

[216] Prabhudesai S., Sinha S., Attar A., Kotagiri A., Fitzmaurice A.G., Lakshmanan R., Ivanova M.I., Loo J.A., Klarner F.G., Schrader T. (2012). A novel “molecular tweezer” inhibitor of alpha-synuclein neurotoxicity in vitro and in vivo. Neurotherapeutics.

[217] Kurnik M., Sahin C., Andersen C.B., Lorenzen N., Giehm L., Mohammad-Beigi H., Jessen C.M., Pedersen J.S., Christiansen G., Petersen S.V. (2018). Potent alpha-synuclein aggregation inhibitors, identified by high-throughput screening, mainly target the monomeric state. Cell Chem. Biol..

[218] Uversky V.N. (2015). Intrinsically disordered proteins and their (disordered) proteomes in neurodegenerative disorders. Front. Aging Neurosci..

[219] Schweighauser M., Shi Y., Tarutani A., Kametani F., Murzin A.G., Ghetti B., Matsubara T., Tomita T., Ando T., Hasegawa K. (2020). Structures of alpha-synuclein filaments from multiple system atrophy. Nature.

[220] Guerrero-Ferreira R., Taylor N.M., Arteni A.A., Kumari P., Mona D., Ringler P., Britschgi M., Lauer M.E., Makky A., Verasdonck J. (2019). Two new polymorphic structures of human full-length alpha-synuclein fibrils solved by cryo-electron microscopy. eLife.

[221] Shahnawaz M., Mukherjee A., Pritzkow S., Mendez N., Rabadia P., Liu X., Hu B., Schmeichel A., Singer W., Wu G. (2020). Discriminating alpha-synuclein strains in Parkinson’s disease and multiple system atrophy. Nature.

[222] Ryan P., Patel B., Makwana V., Jadhav H.R., Kiefel M., Davey A., Reekie T.A., Rudrawar S., Kassiou M. (2018). Peptides, peptidomimetics, and carbohydrate–peptide conjugates as amyloidogenic aggregation inhibitors for Alzheimer’s disease. ACS Chem. Neurosci..

[223] Azzarito V., Long K., Murphy N.S., Wilson A.J. (2013). Inhibition of α-helix-mediated protein-protein interactions using designed molecules. Nat. Chem..

[224] Sonti R., Gopi H.N., Muddegowda U., Ragothama S., Balaram P. (2013). A designed three-stranded β-sheet in an α/β hybrid peptide. Chemistry.

[225] Wrasidlo W., Tsigelny I.F., Price D.L., Dutta G., Rockenstein E., Schwarz T.C., Ledolter K., Bonhaus D., Paulino A., Eleuteri S. (2016). A de novo compound targeting α-synuclein improves deficits in models of Parkinson’s disease. Brain.

[226] Price D.L., Koike M.A., Khan A., Wrasidlo W., Rockenstein E., Masliah E., Bonhaus D. (2018). The small molecule alpha-synuclein misfolding inhibitor, NPT200-11, produces multiple benefits in an animal model of Parkinson’s disease. Sci. Rep..

[227] Levenson J.M., Schroeter S., Carroll J.C., Cullen V., Asp E., Proschitsky M., Gannon K.S. (2016). NPT088 reduces both amyloid-β and tau pathologies in transgenic mice. Alzheimers Dement. TRCI.

[228] Gordon D.J., Tappe R., Meredith S.C. (2002). Design and characterization of a membrane permeable N-methyl amino acid-containing peptide that inhibits Abeta1-40 fibrillogenesis. J. Pept. Res..

[229] Adessi C., Frossard M.-J., Boissard C., Fraga S., Bieler S., Ruckle T., Vilbois F., Robinson S.M., Mutter M., Banks W.A. (2003). Pharmacological profiles of peptide drug candidates for the treatment of Alzheimer’s disease. J. Biol. Chem..

[230] Stott K. (2001). Peptides Containing N-substituted L-amino Acids for Preventing Beta-Strand Association. https://patents.google.com/patent/WO2001007473A1/en.

[231] Ruzza P., Gazziero M., De Marchi M., Massalongo G., Marchiani A., Autiero I., Tessari I., Bubacco L., Calderan A.P. (2015). Peptides as modulators of α-synuclein aggregation. Protein Pept. Lett..

[232] Szegő E.M., Boß F., Komnig D., Gärtner C., Höfs L., Shaykhalishahi H., Wördehoff M.M., Saridaki T., Schulz J.B., Hoyer W. (2021). A β-Wrapin targeting the N-terminus of α-synuclein monomers reduces fibril-induced aggregation in neurons. Front. Neurosci..

[233] Rodriguez J.A., Ivanova M.I., Sawaya M.R., Cascio D., Reyes F.E., Shi D., Sangwan S., Guenther E.L., Johnson L.M., Zhang M. (2015). Structure of the toxic core of α-synuclein from invisible crystals. Nature.

[234] Sangwan S., Sahay S., Murray K.A., Morgan S., Guenther E.L., Jiang L., Williams C.K., Vinters H.V., Goedert M., Eisenberg D.S. (2020). Inhibition of synucleinopathic seeding by rationally designed inhibitors. eLife.

[235] Giacomelli C., Daniele S., Martini C. (2017). Potential biomarkers and novel pharmacological targets in protein aggregation-related neurodegenerative diseases. Biochem. Pharmacol..

[236] Lie P.P., Nixon R.A. (2019). Lysosome trafficking and signaling in health and neurodegenerative diseases. Neurobiol. Dis..

[237] Bonam S.R., Wang F., Muller S. (2019). Lysosomes as a therapeutic target. Nat. Rev. Drug Discove..

[238] Assêncio F.R. (2019). Alpha-synuclein as therapeutic target in Parkinson’s disease. Neuroforum.

[239] Fakhree M.A.A., Konings I.B.M., Kole J., Cambi A., Blum C., Claessens M.M.A.E. (2021). The localization of alpha-synuclein in the endocytic pathway. Neuroscience.

[240] Cuervo A.M., Dice J.F., Fredenburg R., Lansbury P.T., Sulzer D. (2004). Impaired degradation of mutant α-synuclein by chaperone-mediated autophagy. Science.

[241] Mazzulli J.R., Zunke F., Isacson O., Studer L., Krainc D. (2016). α-synuclein-induced lysosomal dysfunction occurs through disruptions in protein trafficking in human midbrain synucleinopathy models. Proc. Natl. Acad. Sci. USA.

[242] Scheidt T., Łapińska U., Kumita J.R., Whiten D.R., Klenerman D., Wilson M.R., Chen S.A.I., Linse S., Vendruscolo M., Dopson C.M. (2019). Secondary nucleation and elongation occur at different sites on Alzheimer’s amyloid—Aggregates. Sci. Adv..

[243] Shin Y., Klucken J., Patterson C., Hyman B.T., McLean P.J. (2005). The co-chaperone carboxyl terminus of Hsp70-interacting protein (CHIP) mediates alpha-synuclein degradation decisions between proteasomal and lysosomal pathways. J. Biol. Chem..

[244] Luk K.C., Mills I.P., Trojanowski J.Q., Lee V.M. (2008). Interactions between Hsp70 and the hydrophobic core of alpha-synuclein inhibit fibril assembly. Biochemistry.

[245] Danzer K.M., Ruf W.P., Putcha P., Joyner D., Hashimoto T., Glabe C., Hyman B.T., McLean P.J. (2011). Heat-shock protein 70 modulates toxic extracellular alpha-synuclein oligomers and rescues trans-synaptic toxicity. FASEB J..

[246] Kalia L.V., Kalia S.K., Chau H., Lozano A.M., Hyman B.T., McLean P.J. (2011). Ubiquitinylation of alpha-synuclein by carboxyl terminus Hsp70-interacting protein (CHIP) is regulated by Bcl-2-associated athanogene 5 (BAG5). PLoS ONE.

[247] Hu S., Tan J., Qin L., Lv L., Yan W., Zhang H., Tang B.S., Wang C. (2021). Molecular chaperones and Parkinson’s disease. Neurobiol. Dis..

[248] Klucken J., Shin Y., Masliah E., Hyman B., Mclean P. (2004). Hsp70 reduces alpha-synuclein aggregation and toxicity. J. Biol. Chem..

[249] Jia C., Ma X., Liu Z., Gu J., Zhang X., Li D., Zhang S. (2019). Different heat shock proteins bind α-synuclein with distinct mechanisms and synergistically prevent its amyloid aggregation. Front. Neurosci..

[250] Jones D.R., Moussaud S., McLean P. (2014). Targeting heat shock proteins to modulate α-synuclein toxicity. Ther. Adv. Neurol. Disor..

[251] Cullen V., Lindfors M., Ng J., Paetau A., Swinton E., Kolodziej P., Boston H., Saftig P., Woulfe J., Feany M.B. (2009). Cathepsin D expression level affects alpha-synuclein processing, aggregation, and toxicity in vivo. Mol. Brain..

[252] Colacurcio D.J., Nixon R.A. (2016). Disorders of lysosomal acidification—The emerging role of v-ATPase in aging and neurodegenerative disease. Ageing Res. Rev..

[253] Stefanis L., Emmanouilidou E., Pantazopoulou M., Kirik D., Vekrellis K., Tofaris G.K. (2019). How is alpha-synuclein cleared from the cell?. J. Neurochem..

[254] Masliah E., Rockenstein E., Mante M., Crews L., Spencer B., Adame A., Patrick C., Trejo M., Ubhi K., Rohn T.T. (2011). Passive immunization reduces behavioral and neuropathological deficits in an alpha-synuclein transgenic model of Lewy body disease. PLoS ONE.

[255] Schenk D.B., Koller M., Ness D.K., Griffith S.G., Grundman M., Zago W., Soto J., Atiee G., Ostrowitzki S., Kinney G.G. (2017). First-in-human assessment of PRX002, an anti-α-synuclein monoclonal antibody, in healthy volunteers. Mov. Disord..

[256] Games D., Valera E., Spencer B., Rockenstein E., Mante M., Adame A., Patrick C., Ubhi K., Nuber S., Sacayon P. (2014). Reducing C-terminal-truncated alphasynuclein by immunotherapy attenuates neurodegeneration and propagation in Parkinson’s disease-like models. J. Neurosci..

[257] Weihofen A., Liu Y., Arndt J.W., Huy C., Quan C., Smith B.A., Baeriswyl J.-L., Cavegn N., Senn L., Su L. (2019). Development of an aggregate-selective, human-derived α-synuclein antibody BIIB054 that ameliorates disease phenotypes in Parkinson’s disease models. Neurobiol. Dis..

[258] Messer A., Butler D.C. (2019). Optimizing intracellular antibodies (intrabodies/nanobodies) to treat neurodegenerative disorders. Neurobiol. Dis..

[259] Lynch S.M., Zhou C., Messer A. (2008). An scFv intrabody against the nonamyloid component of alpha-synuclein reduces intracellular aggregation and toxicity. J. Mol. Biol..

[260] El Turk F., De Genst E., Guilliams T., Fauvet B., Hejjaoui M., Di Trani J., Chiki A., Mittermaier A., Vendruscolo M., Lashuel H.A. (2018). Exploring the role of post-translational modifications in regulating alpha-synuclein interactions by studying the effects of phosphorylation on nanobody binding. Protein Sci..

[261] Nimmo J.T., Verma A., Dodart J.-C., Wang C.Y., Savistchenko J., Melki R., Carare R.O., Nicoll J.A.R. (2020). Novel antibodies detect additional α-synuclein pathology in synucleinopathies: Potential development for immunotherapy. Alzheimers Res. Ther..

[262] Volc D., Poewe W., Kutzelnigg A., Lührs P., Thun-Hohenstein C., Schneeberger A., Galabova G., Majbour N., Vaikath N., El-Agnaf O. (2020). Safety and immunogenicity of the α-synuclein active immunotherapeutic PD01A in patients with Parkinson’s disease: A randomised, single-blinded, phase 1 trial. Lancet Neurol..

[263] Mandler M., Valera E., Rockenstein E., Weninger H., Patrick C., Adame A., Santic R., Meindl S., Vigl B., Smrzka O. (2014). Next-generation active immunization approach for synucleinopathies: Implications for Parkinson’s disease clinical trials. Acta Neuropathol..

[264] Rockenstein E., Ostroff G., Dikengil F., Rus F., Mante M., Florio J., Adame A., Trinth I., Kim C., Overak C. (2018). Combined active humoral and cellular immunization approaches for the treatment of synucleinopathies. J. Neurosci..

[265] Mao X., Ou M.T., Karuppagounder S.S., Kam T.I., Yin X., Xiong Y., Ge P., Umanah G.E., Brahmachari S., Shin J.H. (2016). Pathological α-synuclein transmission initiated by binding lymphocyte-activation gene 3. Science.

[266] Kim S., Yun S.P., Lee S., Umanah G.E., Bandaru V.V.R., Yin X., Rhee P., Karuppagounder S.S., Kwon S.H., Lee H. (2018). GBA1 deficiency negatively affects physiological α-synuclein tetramers and related multimers. Proc. Natl. Acad. Sci. USA.

[267] Parkinson’s Disease: Challenges, Progress, and Promise (2016). NIH Publication No.15-5595. https://www.ninds.nih.gov/Disorders/All-Disorders/Parkinsons-Disease-Challenges-Progress-and-Promise.

